# Minimum Requirements for Taxicab Security Cameras^*^

**DOI:** 10.4236/jtts.2014.43022

**Published:** 2014-07

**Authors:** Shengke Zeng, Harlan E. Amandus, Alfred A. Amendola, Bradley H. Newbraugh, Douglas M. Cantis, Darlene Weaver

**Affiliations:** Division of Safety Research, National Institute for Occupational Safety and Health, Centers for Disease Control and Prevention, Morgantown, WV, USA

**Keywords:** Taxicab Homicide, Facial Identification, Photographic Resolution, Dynamic Range, Lens Distortion, Motion Blur

## Abstract

**Problem:**

The homicide rate of taxicab-industry is 20 times greater than that of all workers. A NIOSH study showed that cities with taxicab-security cameras experienced significant reduction in taxicab driver homicides.

**Methods:**

Minimum technical requirements and a standard test protocol for taxicab-security cameras for effective taxicab-facial identification were determined. The study took more than 10,000 photographs of human-face charts in a simulated-taxicab with various photographic resolutions, dynamic ranges, lens-distortions, and motion-blurs in various light and cab-seat conditions. Thirteen volunteer photograph-evaluators evaluated these face photographs and voted for the minimum technical requirements for taxicab-security cameras.

**Results:**

Five worst-case scenario photographic image quality thresholds were suggested: the resolution of XGA-format, highlight-dynamic-range of 1 EV, twilight-dynamic-range of 3.3 EV, lens-distortion of 30%, and shutter-speed of 1/30 second.

**Practical Applications:**

These minimum requirements will help taxicab regulators and fleets to identify effective taxicab-security cameras, and help taxicab-security camera manufacturers to improve the camera facial identification capability.

## 1. Introduction

Workplace violence has consistently been a leading cause of workplace fatalities and injuries since national occupational health surveillance efforts began at the National Institute for Occupational Safety and Health (NIOSH) in 1980 [1]. The latest preliminary data available from the Bureau of Labor Statistics reveals that in 2011 there were 458 homicides, making homicides the fourth leading cause of work-related fatalities [2]. There were 32 homicides in the Taxi Service Sector in 2011 [3]. Taxicab drivers, within the transportation industry, were more likely to be victims of homicide than workers of any industry (7.4 per 100,000 workers), 20 times greater than that of all workers (0.37 per 100,000 workers) [4].

The epidemiology of safety risks for taxicab drivers is incomplete and scattered; however, the subject is discussed briefly in several summary research articles [5]–[10]. Because available literature focusing on the epidemiology of workplace violence among taxicab drivers is limited; very little is known conclusively about workplace violence risk factors in this industry.

In 2000, the Occupational Safety and Health Administration (OSHA) made the following recommended safety measures for taxicab drivers: 1) utilizing global positioning systems (GPS) to locate a driver in distress, 2) incorporating caller ID to help trace client location, 3) carrying first-aid kits, 4) using in-car surveillance cameras, 5) installing partitions, 6) establishing a protocol with police, 7) using radios for emergency communication, 8) enrolling in safety training, 9) employing silent alarms as a danger alert, and 10) using cashless fare systems for payment [11].

Among these OSHA safety recommendations, many taxicab industry stakeholders favor taxicab security cameras as a preferred intervention because they provide the perception of surveillance to the potential perpetrators (thereby functioning as a deterrent) while also increasing the arrest rate (another relevant outcome) of perpetrators. Some cities have already been consulting with safety experts and installing security cameras in their taxicabs as a deterrent for crimes against taxicab drivers. These cities include Las Vegas, San Francisco, Seattle, Toronto, Vancouver, Winnipeg, Dallas, Houston, Orlando, Portland, San Antonio and New Orleans [12].

NIOSH has recently completed an epidemiologic study that suggests that taxicab security camera systems are highly effective in reducing taxicab driver deaths [4]. The findings from this study showed that the cities with taxicab security cameras experienced a threefold reduction in taxicab driver homicides compared with control cities. Other reports also showed that fitting a camera in taxicabs led to a reduction in incidents of abuse toward taxi drivers [13], and there was a reduction in fare jumpers and that rowdy passengers were more subdued when in a taxicab with a camera system [14]. While there is general agreement that taxicab security cameras are effective in reducing violent crime against taxicab drivers, there has been currently no peer-reviewed published literature evaluating the technical effectiveness of taxicab security camera models in use in the United States for safety or crime deterrence. Also, there is no national taxicab security camera selection guidance in the United States. Only a few domestic and international cities did issue local taxicab security camera regulations or guidance [15]–[20], or a study did report on taxi industry safety and security [21]. These local regulations and report specified individual regulatory taxicab security camera system requirement metrics and some recommendations, but did not provide the engineering methodologies, procedures and evaluations to determine these metrics.

Given the acknowledged effectiveness of in-cab surveillance systems in reducing violent crime against taxi drivers, acknowledging the lack of standardization in dedicated systems for this purpose and engineering research to determine minimally acceptable requirements for in-taxi surveillance and facial identification are needed. In order to develop these minimum technical requirements, NIOSH has recently completed engineering studies to establish a protocol for determining taxicab security camera performance criteria.

The objective of the current study was to quantify the minimum technical requirements for taxicab security camera systems to perform in-taxicab passenger’s facial identification under various in-taxicab light conditions and seat positions. This study did the followings to quantify the minimum requirements: 1) recorded actual lighting environments of taxicabs by measuring various real open-field light conditions in various seats in a simulated taxicab; 2) used artificial lights to reconstruct these light conditions in the same seats of the simulated taxicab; 3) photographed human subject faces in the taxicab with common and infrared cameras in these simulated taxicab lighting environments in various focal lengths, exposures, and shutter speeds; 4) constructed sets of human facial images with different resolutions, dynamic ranges, lens distortions, and motion blurs in different light conditions and seat positions; 5) sent these sets of human facial images to experienced photo evaluators for minimum acceptable threshold determination by voting; and 6) made statistical analyses on these threshold votes to determine the minimum requirements for the taxicab security camera systems.

Information on minimum requirements for taxicab security cameras will help taxicab-industry regulatory officials and taxicab fleet owners or managers to identify taxicab security cameras meeting minimum facial identification capabilities, and help taxicab security camera manufacturers improve the facial identification capability of their cameras. This should improve arrest and prosecution of suspects in taxicab crimes. Improving the capability of cameras to identify perpetrators also holds potential for increasing the crime-deterrent effects of the cameras and thus improve taxicab driver safety.

## 2. Methods/Procedures

### 2.1. Overview of Taxicab Security Camera Evaluation

The following terminology will appear in Sections 2, 3, 4, and 5.

Light conditions:

L1—daylight;
L2—moonless dark;
L3—moonless dark with backlight;
L4—sunset via rear window;
Lux—luminous flux per unit area; light intensity unit.

Camera parameters:

RES—photograph resolution, measured by line-widths-per-head height using Imatest software;
MTF—modulation transfer function; a parameter to measure the sharpness of a photographic image;
LPHH—line-widths-per-head height; a photographic resolution unit, measured as the MTF falls 50% from its peak;
DRH—photographic dynamic range in highlight (in right lighting condition), measured by gray steps or exposure value;
DRS—photographic dynamic range in shadow (in twilight condition), measured by gray steps or exposure value;
EV—exposure value; a measure of photographic dynamic range;
LD—lens distortion, measured by percentage (%);
MB—motion blur, measured by millisecond (mS).

Others:

CCD—charge-coupled device; a type of image sensor;
CMOS—complementary metal-oxide-semiconductor; a type of image sensor;
HDR—high-dynamic-range imaging;
LCD—liquid crystal display;
LED—light emitting diode;
VGA—video graphics array; a computer display system with a resolution of 640 by 480 pixels.

A taxicab security camera system usually consists of a camera and an image recorder. The camera, installed in the front of a taxicab, captures still or video images of the activities inside the taxicab. The image recorder, usually installed in the trunk or other concealed space in the taxicab, processes the captured digital images and stores the digital image files onto a flash memory card or hard disk drive.

The camera is the most important element of a taxicab security camera system. Its image quality directly affects the ability of the taxicab security camera system to successfully identify, arrest, and convict a suspect in a crime event [22]. Currently, no quantitative national standards are available in the United States to specify the minimum image resolution and other camera features that affect facial identification capabilities of a taxicab security camera and, likewise, there is no standard test protocol for evaluating security cameras. The taxicab security camera manufacturers typically provide very limited camera specification parameters, such as imaging sensor dimension, sensor pixel-count and/or the bit-depth of the sensor analog-to-digital converter [23]–[25]. Although these optoelectronic parameters of a taxicab security camera are important and will affect taxicab security camera test results, they are not sufficient to specify the camera’s photographic quality. The photographic image quality of a taxicab security camera is the result of the camera’s performance with a combined optoelectronic configuration (including sensor dimension, sensitivity, resolution, and noise figure; camera focus, shutter speed, and aperture; and lens view-angle, transmittance, and distortion) in various cab-light conditions and geometric cab-seat positions.

To comprehensively assess a taxicab security camera’s photographic quality, this study proposed five photographic-image quality metrics to evaluate an in-cab face photograph taken by the camera: 1) photographic resolution, 2) highlight dynamic range, 3) shadow dynamic range, 4) lens distortion, and 5) motion blur. These metrics were developed to account for the unique circumstances of in-taxicab facial identification. The study quantified the minimum requirements of these metrics under various in-cab light conditions and seat positions. With these minimum requirements, an in-cab face photograph may contain enough facial information for customer face identification. These minimum requirements constitute the thresholds that ensure the minimum acceptable image quality of a taxicab security camera which would be capable of customer facial identification.

To determine the thresholds of these photographic-image quality parameters, a color reference camera (Nikon D5000, Nikon Corporation, Tokyo, Japan) with a complementary metal-oxide-semiconductor (CMOS) image sensor and a black-and-white reference camera with a charge-coupled device (CCD) image sensor, which has infrared sensing capability (GRAS-14S5M-C, Point Grey, Richmond, British Columbia, Canada), were used to photograph photograph-test charts [26] with 10 human subject facial image charts on them, in four extreme cab-light conditions: 1) daylight (L1), 2) moonless dark (L2), 3) moonless dark with backlight (L3), and 4) sunset via rear window (L4). (The color camera without infrared capability can operate only in L1 and L4.). Each cab-light condition was also photographed in three different cab-seat positions: 1) front-right, 2) rear-right, and 3) rear-middle seats. During photographing, the camera’s sensitivity, focus, aperture, and shutter speed were adjusted to yield in-cab face photographs with various degrees of reduced photographic resolution, highlight dynamic range, shadow dynamic range, and various degrees of deteriorated motion blur. The different degrees of lens distortion of face photographs were altered by using image-editing software (Photoshop CS5, Adobe Systems, San Jose, California). These in-cab facial photographs were sorted and sent to 13 volunteer photograph evaluators for photographic-image quality evaluation. The photograph evaluators examined the face photographs and determined by voting the thresholds of five photographic-image quality parameters in each of four in-cab light conditions and three seat positions. The five statistical thresholds are the minimum technical requirements for a taxicab security camera.

In order to maintain the consistency of the human facial photographic image quality in various lighting conditions and seating positions, the reference cameras photographed human subject facial image charts, instead of real human subject faces, in the photographic parameter threshold determinations. The difference between the photograph of a two-dimensional (2-D) facial chart and the photograph of a three-dimensional (3-D) human face is the different facial shadow pattern caused by different geometric light source configurations. Since the light source inside a typical taxicab contains multiple lights from the windshield, side and rear windows, the facial shadows would be mostly reduced by the lights from different directions. The original facial chart photographs were also taken with multiple light sources to further reduce the facial shadow difference. Therefore, the difference between the photograph of a 2-D facial chart and the photograph of a 3-D human face would be mostly reduced by multiple light sources, and would not be considered to affect photograph image consistency. The original facial chart photographs were taken in an ideal lighting condition. As the high-quality reference cameras photograph the 2-D facial charts, the resultant photographs will have the same level of image quality as the photographs of 3-D correspondent human face in ideal light condition, and have the same reduced photographic image quality in deteriorated light conditions.

### 2.2. Quantifying Taxicab Security Camera Performance

#### Five photographic-image quality parameters

Photographic resolution (RES) is one of the important photographic-image quality indicators. The higher the resolution, the more detailed the photograph, and more lines can be observed on a photographed human face. In this study, the photographic resolution is measured by line-widths per head-height to normalize the captured face photographic resolution. Photographic dynamic range is the difference between the lightest and darkest elements on a captured photographic image. The exposure value (EV) unit was used to indicate the level of dynamic range [27]. Photographic dynamic range in highlight (DRH) measures the ability of a taxicab security camera to detect highlight details in a captured photographic image in a bright lighting condition. Photograph dynamic range in shadow (DRS) measures the ability of a taxicab security camera to detect shadow details in a captured photographic image in a twilight lighting condition [28] [29]. Lens distortion (LD) measures the degree of camera lens distortion in percentage. Most single-lens taxicab security cameras have some degree of barrel distortion. Wide-angle lenses often suffer particularly badly from barrel distortion [30]. Motion blur (MB) is caused by slow camera shutter speeds. This study determined the slowest shutter speed at which the degree of motion blur does not severely affect in-cab face identification.

#### Simulated taxicab and three in-cab seat positions

The taxicab security camera evaluation was conducted inside a simulated taxicab that was a retired Ford Crown Victoria Police Interceptor. The vehicle had five seat positions. Since the two front seats and two rear side seats were symmetrical, the camera evaluation was conducted only in three seat positions: front-right, rear-right, and rear-middle.

#### Four in-cab light conditions

This study categorized the in-cab light conditions into four levels: daylight (L1; 1000 – 7000 lux, depending on seat positions and light meter orientation in the seat) [31], moonless dark (L2; 0 – 2 lux), moonless dark with backlight by automobile headlights via rear window (L3; 1 – 500 lux, depending on whether the light meter faces toward the headlights), and sunset via rear window (L4; 400 – 8000 lux, depending on whether the light meter faces toward the sunrays). The contrast between L1 and L2 is very high. To take properly exposed photographs in both L1 and L2 conditions, a camera should have an infrared light source and automatic shutter speed/aperture control. L3 is a difficult light condition for cameras with an infrared sensor facing a rear window; headlights of vehicles could lure the infrared sensor to shut off the infrared LEDs, causing the camera to operate without infrared LED illumination. In L4 condition, a camera would directly face bright sunrays through the rear window, and the camera could likely yield some partially washed-out in-cab photographs.

**Figure 1:**
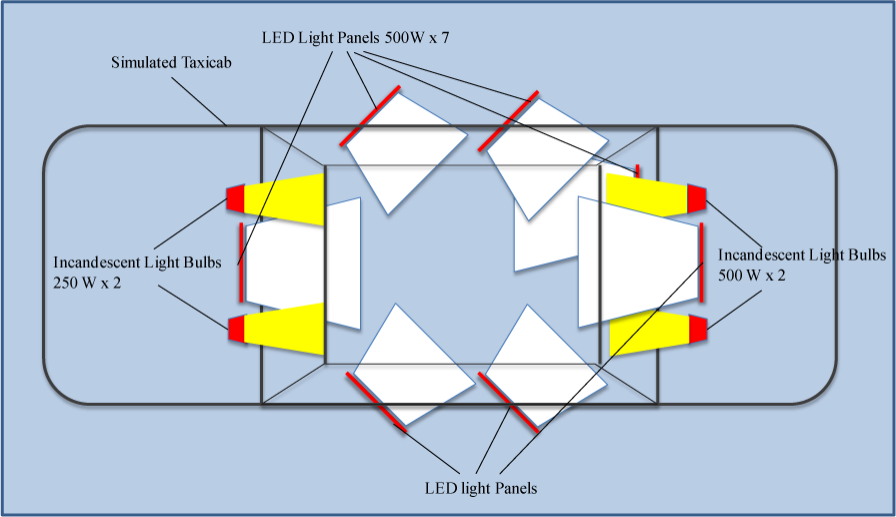
Schematics of reconstruction of light conditions in a taxicab with seven white LED light panels and four incandescent light bulbs. (All of the figures and tables are 2-column fitting).

**Figure 2:**
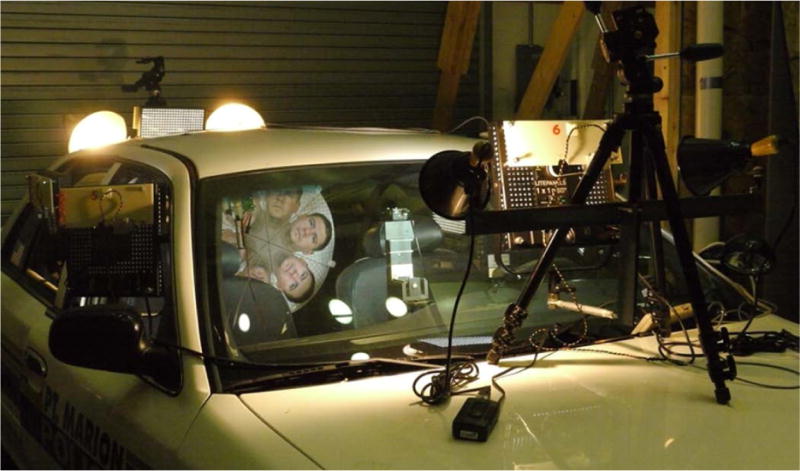
Four light conditions in each of the three taxicab seats were reconstructed in a simulated taxicab with seven white LED light panels and four incandescent light bulbs.

The in-cab light measurements in the open field with real sunlight, darkness, and headlight backlight conditions were necessary to determine the practical light conditions inside a taxicab in the above four light conditions. A handheld light meter (Mavolux 5032B, Gossen, Hamburg, Germany) was used to measure the illumination levels (lux) and a handheld light color temperature meter (Kenko KCM-3100, Kenko Co., LTD., Tokyo, Japan) was used to measure the light color temperature (K) in the three seat positions in the simulated taxicab in the above four light conditions. During the taxicab security camera evaluation, these four light conditions were reconstructed in the simulated taxicab in a NIOSH lab using seven white LED light panels with quartz-halogen equivalent 500 W light output and 5600 K light color temperature (LITE-PANELS 1 × 1′ LED 5600 K Daylight Super-Spot/Reg, Litepanels US, Van Nuys, California). Four incandescent tungsten light bulbs (2 × 250 W in front of the windshield and 2 × 500 W outside the rear window) with low color temperature are mixed with white LED light in L4 light simulation to reduce the total light color temperature to near the level of sunset color temperature. Figure 1 shows the schematic of the light simulation setup, and Figure 2 shows the light condition reconstruction setup in a simulated taxicab with LED light panels and incandescent light bulbs.

#### Resolution thresholds

**Figure 3:**
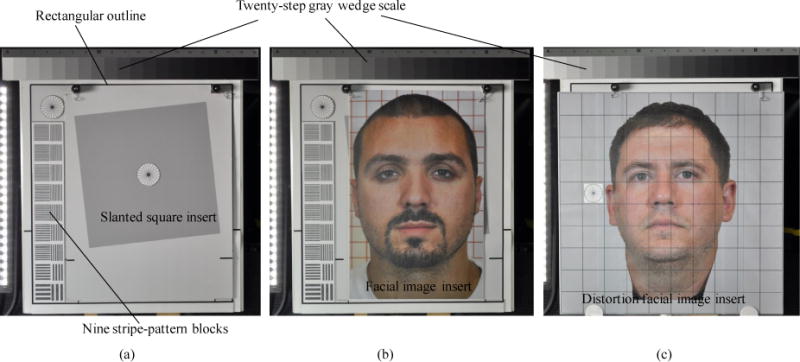
The photo test chart contains a slanted square insert [shown in (a)], 10 human subject facial image inserts [one of the inserts is shown in (b)], 10 distortion facial image inserts [one of the inserts is shown in (c)], a 20-step gray step scale, 9 stripe-pattern blocks, and a square outline. The size of the outline is 30 cm × 30 cm.

A photograph-test chart was used in taxicab security camera evaluations to determine photographic resolution, dynamic range thresholds and lens distortion. The test chart contains a slanted square image inset, 10 human subject facial image insets, 9 stripe pattern blocks, and a 30 cm × 27.5 cm rectangular outline for resolution measurement. On the top of the test chart there is a 20-step gray wedge scale (14-inch Kodak Q-14 Gray Scale Chart, Eastman Kodak Company, Rochester, New York) for dynamic range measurement. The test chart also contains 10 gridded distortion facial image insets for lens distortion measurement. The test chart and its components are shown in Figure 3.

**Figure 4:**
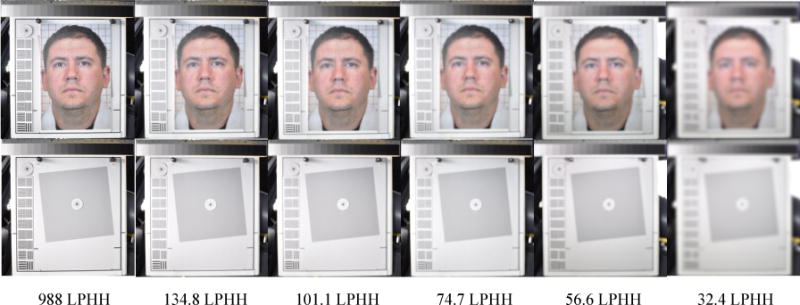
Six facial photo/slanted square pairs with various resolutions from 988 to 32 LPHH. Each pair of the facial photo and slanted square shares the same resolution.

The photographic resolution is determined by measuring the modulation transfer function (MTF) of the slanted square image using image quality test software Imatest Master (Imatest LLC, Boulder, Colorado). The MTF is the spatial frequency response of an imaging system. It is a commonly used metric for defining the spatial resolution characteristics of imaging systems. The Imatest software determines image resolution by measuring the MTF of a slanted-edge square target [32]. The software could not directly determine the photographic resolution of human facial images. It determined the facial image resolution by measuring the MTF of the corresponding slanted square image inset. During the MTF determination, the slanted square image and 10 facial images were inserted into the rectangular outline on the test chart and photographed by a reference camera one-by-one. The reference camera photographed the slanted square and 10 facial image insets with the same focal length, so that the 11 slanted-square/facial photographs share the same resolution. During the resolution measurement, the Imatest software recorded the MTF of the slanted square image varying from high to low versus the spatial frequency varying from low to high. The software defines the image resolution as the spatial frequency where the MTF value falls 50% from its peak. The spatial frequency unit (also the resolution unit) is line-widths per picture-height. The picture height here was the height of the rectangular outline. The outline height (30 cm) was higher than any of the 10 facial image insets whose head-heights varied from 22.2 to 25.5 cm. Since the picture height here was the height of a simulated human head (the outline height, 30 cm), the image resolution unit in this MTF measurement could be replaced with line-widths per head-height (LPHH). The LPHH unit normalizes the resolution of a captured facial image taken either in a front or back seat. With the same LPHH, no matter where the customer sat, their facial images would have the same resolution. During photograph resolution threshold determination, a reference camera photographed the test chart with the slanted square image inset and facial image insets with six altered focal lengths that yielded photographs with six different photograph resolutions, from the sharpest (400 – 1000 LPHH) to the blurriest (25 – 35 LPHH), depending on the focal length, which was controlled by the stripe pattern blocks on the left-hand side of the chart (Figure 4).

The resolution photograph taking was repeated in four light conditions and three seat positions. The facial photographs with different resolutions were sent to photograph evaluators for a resolution threshold vote. After facial photographs with minimum acceptable sharpness in a specific light/seat condition were voted on by the evaluators, the resolution of the corresponding slanted square photograph would be measured using Imatest software, and the resultant LPHH resolution would be the resolution threshold in this light/seat condition.

#### Dynamic range thresholds

**Figure 5:**
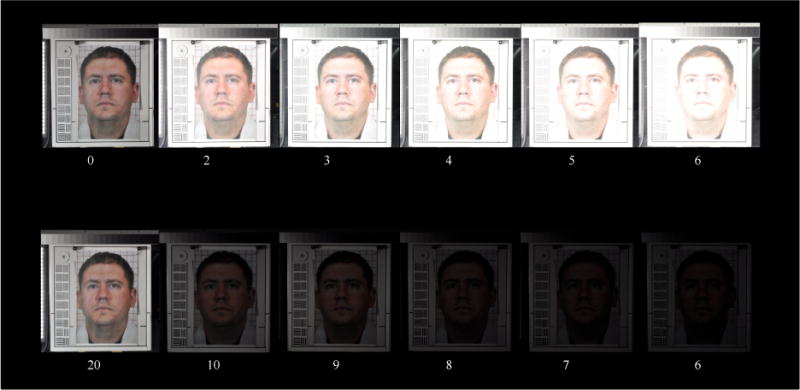
Six facial photos with different DRH (first row) with the number of washed-out gray steps, and six facial photos with different DRS (second row) with the number of recognizable gray steps.

The photograph-test chart contains a 14-inch Kodak Q-14 Gray Scale Chart, which has 20 gray steps, with 1/3 EV per step, on the top of the chart (Figure 3). The gray scale chart was used to measure the DRH and DRS of a facial photograph. The total dynamic range covered by these gray steps is 6.7 EV, which is wide enough to measure the dynamic range of a face photograph taken by a typical taxicab security camera. A taxicab security camera with more DRH can detect more detail in highlight condition, and a taxicab security camera with more DRS will detect more details in twilight condition. This gray scale chart can measure a camera’s DRH and DRS with an accuracy of 1/3 EV. During DRH measurement, the shutter speed and aperture of a reference camera were adjusted to photograph the photograph-test chart with each of 10 facial image insets with six exposure levels ranging from normal to five different degrees of overexposure. The normally exposed photograph shows most of the gray steps of the Gray Scale Chart. The five overexposed photographs showed two, three, four, five, and six washed-out gray steps, which reduces the highlight dynamic range. During DRS measurement, the shutter speed and aperture of a reference camera were adjusted to photograph the photograph-test chart with each of 10 facial image insets with six exposure levels ranging from normal to five different degrees of underexposure. The normally exposed photograph shows most of the gray steps of the Gray Scale Chart. The five underexposed photographs showed only 10, 9, 8, 7, and 6 gray steps, which reduces the shadow dynamic range. The DRH and DRS measurements were repeated with each of the four light and three seat combinations. These DRH and DRS facial photographs were sent to photograph evaluators for DRH/DRS threshold votes. The evaluators voted for the minimum acceptable DRH and DRS photographs in each of the light/seat conditions. The number of washed-out gray steps or the numbers of recognizable gray steps in a selected photograph would be the threshold of DRH or DRS measurement in this light/seat condition, respectively, as shown in Figure 5.

#### Lens distortion thresholds

**Figure 6:**
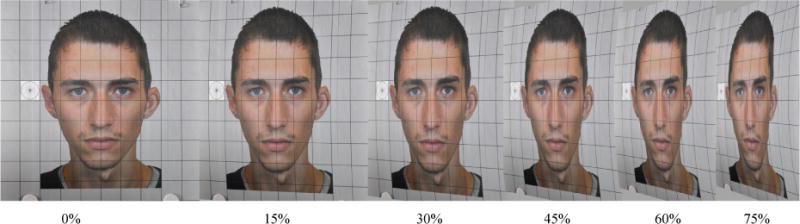
Six facial photos with different percentages of lens distortion.

The lens distortion of the facial photographs was altered using the graphic editing software Photoshop CS5. During lens distortion evaluation, a reference camera photographed each of the 10 gridded facial image insets with the dimension of 30 cm × 30 cm (Figure 3) in each of the four light and three seat conditions. The degree of the lens distortions of the facial photographs were artificially altered by using Photoshop CS5. For each of the light/seat conditions, 5 degrees of lens distorted photographs were altered by the software: 15%, 30%, 45%, 60%, and 75% (Figure 6). The photograph evaluators evaluated the distorted facial photos and voted for the minimum acceptable lens distorted photographs in each of the four light and three seat conditions. The distortion percentages of these acceptable photographs define the lens distortion thresholds in the specific light/seat conditions.

#### Motion blur thresholds

**Figure 7:**
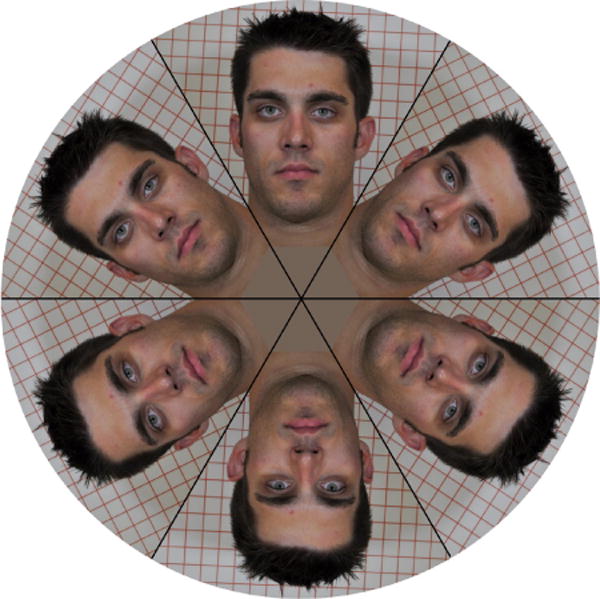
The rotating face board with one of ten human subject facial image pages attached on the board.

The facial photograph of a moving customer is likely to be blurred if it is taken by a taxicab security camera with a slow shutter speed. A blurred facial photograph could deteriorate in-cab human face identification. This study determined shutter speed threshold for motion blur (MB) using a rotating face board. The rotating face board assembly consists of a stepper motor with its shaft attached to the center of a spinning wheel, which is covered with a round facial page attachment with six identical human subject faces on it, as shown in Figure 7. The radius of the spinning wheel is 30 cm.

**Figure 8:**
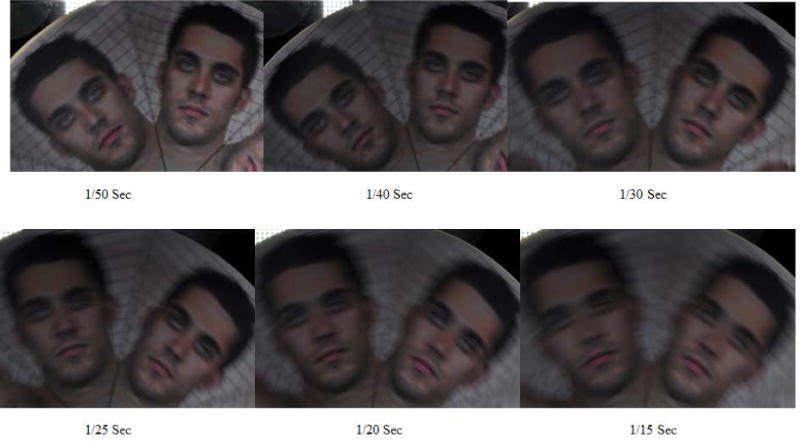
Rotating face board photos taken with 6 different shutter speeds. The facial images become more blurry with slower shutter speeds.

The face images are 60 degrees apart. During shutter threshold determination, the board was installed in the rear-middle seat and spun with a constant angular speed that equaled a common human-head-shaking speed (The head-shaking speed determination is described in “Taxicab security camera Evaluation Procedures”). The rotating faces were photographed by the reference cameras with various shutter speeds: 1/15, 1/20, 1/25, 1/30, 1/40, and 1/50 second in four light conditions. The captured rotating facial photographs become more blurred as the shutter speed becomes slower, as shown in Figure 8. Ten different human subject facial page attachments were photographed by the reference cameras. The photograph evaluators voted for a shutter speed threshold in each of the four light conditions.

#### Infrared LED light source

During the taxicab security camera evaluation, an infrared light source was needed in L2 (dark) and L3 (dark with backlight) conditions. An infrared LED light source was built using nine infrared LEDs with a light wavelength of 870 nm. The light output of the LED light source was measured by a light intensity meter (ILT1700 Research Radiometric/Photometric Light Meter, International Light Technologies, Peabody, Massachusetts). With a distance of 50 cm between the light source and the light intensity sensor, the mean infrared light intensity with 10 measurements was 67.57 μW/cm^2^, which is much lower than the maximum permissible exposure for human eyes to a laser beam with wavelength of 870 nm [33]. A plastic light diffuser was applied in front of the LEDs to have a uniformly distributed infrared light illumination on the photograph-test chart and the rotating face board in L2 and L3 light conditions.

### 2.3. Taxicab Security Camera Evaluation Procedures

**Figure 9:**
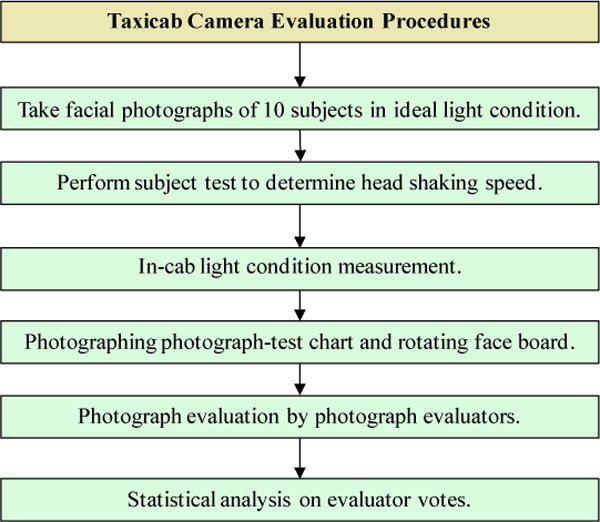
Flowchart of the taxicab camera evaluation procedures.

Figure 9 shows the flow chart of the taxicab security camera evaluation procedures.

#### 1) Take facial photographs of 10 subjects in ideal light condition

The original facial photographs of 10 human subjects were taken by the color reference camera in an ideal light condition. These 10 original face photographs were used to construct 10 facial image insets and 10 gridded distortion facial image insets on the photograph-test chart (Figure 3) and to construct 10 facial image page attachments on the rotating face boards for motion blur (MB) shutter speed threshold determination (Figure 7). To photograph these human faces, 10 male human subjects with ages from 18 to 50 were recruited to simulate taxicab customers. The subject ethnic composition consisted of six white Americans, two African Americans and two Asian Americans. The original facial photographs were taken by the color reference camera to ensure that the image quality would be better than that of any market-available taxicab security cameras. These original facial photographs have much higher photograph resolution and much wider photograph dynamic range than those of any taxicab security cameras.

#### 2) Perform subject test to determine head shaking speed

**Figure 10:**
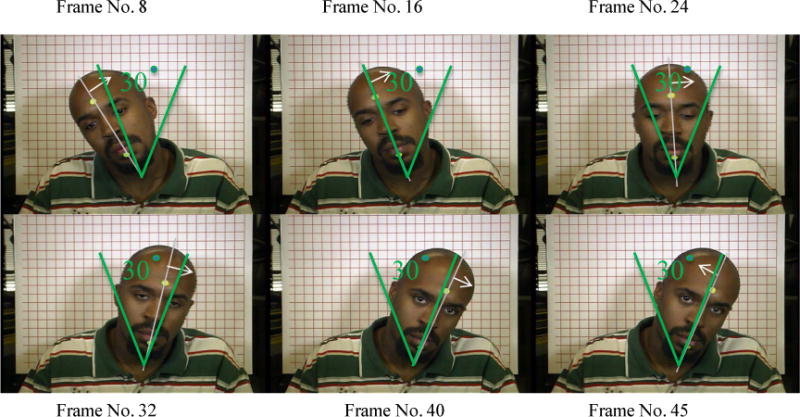
Counting the number of video frames within 30 degrees of head shaking in order to calculate head shaking speed.

To simulate a human head movement in the MB threshold determination, the angular speed of the rotating face board should be equal to common human head-shaking speed. The common human head-shaking speed was determined by human subjects. In the experiment, each of 10 subjects were asked to shake their head around the neck from left to right and back for 50 times at a natural speed controlled by the subject. The head movements were videotaped by a Canon XL1 Mini DV professional camcorder with a frame rate of 29.97 frames per second. In videotape analyses, the angular speed of the head movement was measured by counting the number of video frames within the middle 30 degrees of the head movement as shown in Figure 10.

The head movement angular speed equals 30 degrees divided by the number of frames, multiplied by 29.97 frames per second, where 29.97 frames per second is the video frame rate. The angular speed of each subject’s head movement was the mean of 100 head-shake measurements. The final angular speed of the head movement was the mean of 10 subjects’ head angular speeds.

#### 3) In-cab light condition measurement

In-cab light conditions were measured in the simulated taxicab in the open field. A handheld light meter and light color temperature meter were used to measure the light intensity and light color temperature in the front-right, rear-middle, and rear-right seats. In each seat, the light meter measured the light intensity (lux) in each of the forward, left, right, and rear directions. The light color temperature meter measured the light color temperature in forward direction. The L1 (daylight) condition was measured in a sunny cloudless day at noon time. The L2 (dark) and L3 (dark with backlight) conditions were measured in a moonless night after the afterglow totally disappeared in the sky. During the L3 condition measurement, the headlight beams from a midsized car were projected to the taxicab through the rear window. The L4 (sunset via rear window) condition was measured in a sunny cloudless day at sunset. The sunrays went into the taxicab through the rear window. The measurements were repeated five times in each of the lighting and seat conditions.

#### 4) Photographing photograph-test chart and rotating face board

The photograph-test chart with a slanted square inset, 10 subject facial image insets and 10 gridded subject facial image insets, and the rotating face board with 10 attached subject facial image pages were photographed by reference cameras to determine the thresholds of five photographic parameters in four light conditions and three seat positions. In each of three cab seat positions and each of four light conditions, the light intensities and colors were reconstructed in the simulated taxicab before photographing. During light condition reconstruction, a LabVIEW computer program, developed in-house, controlled the output of the seven LED light panels so that the light intensities in each seat in four directions (forward, left, right, and backward) were within 10% of the in-taxicab light conditions that were measured in the open field. The handheld light meter was used to examine the light intensity. The light color temperature meter was used to monitor the light color temperature. In L4 light condition reconstruction, four incandescent tungsten light bulbs were used to lower the in-cab light color temperature to the approximate color temperature of the sunset. The color reference camera was used to simulate color taxicab security cameras to photograph the photograph-test charts and rotating face board in L1 and L4 light conditions. The black-and-white, infrared reference camera photographed the same chart and rotating board in L1, L2, L3, and L4 light conditions to simulate black-and-white taxicab security cameras with infrared capability. For each test chart inset, the reference camera took three pictures in each of the light/seat conditions.

During the photograph-test chart photographing for resolution threshold determination, the focal length of the reference camera was altered to yield facial photographs with five degrees of blurriness from the sharpest to the blurriest. The degrees of blurriness were controlled by examining the blurriness of the stripe pattern blocks on the left-hand side of the chart during photographing. The 1^st^ to 4^th^ degrees of blurriness were determined as the focal length of the camera was altered until the stripes of the respective 2^nd^, 4^th^, 6^th^, and 9^th^ stripe pattern block looked diffused on the computer LCD display. The 5^th^ degree of blurriness (the blurriest) was obtained by turning the manual focus to a marked position during photographing.

For each degree of blurriness, the reference camera photographed the test chart with the slanted square image inset first, and then photographed the test chart with each of 10 human subject facial image insets one at a time. By doing so, at each of five degrees of blurriness, the 10 subject facial photographic images would have a corresponding slanted square image with the same degree of blurriness (Figure 4). The photographic image resolution of the 10 facial images would be known by testing the resolution of the slanted square image using the Imatest software.

During the photograph-test chart photographing for dynamic range threshold determination, the shutter speed and aperture of the reference camera was altered to yield five various degrees of overexposed facial photographs for DRH measurement. The degree of exposure was controlled by examining the Q-14 Gray Scale on the chart. For taking the 1^st^ to 5^th^ degrees of overexposed photographs, the shutter speed and aperture were altered until two, three, four, five, and six of the left-hand-side gray steps on the gray scale were observed to be washed-out and indistinguishable on the computer display. The shutter speed and aperture of the reference camera was also altered to yield five various degrees of underexposed facial photographs for DRS measurement. For taking the 1^st^ to 5^th^ degrees of underexposed photographs, the shutter speed and aperture were altered until only ten, nine, eight, seven, and six of the left-hand-side gray steps on the gray scale could be recognized on the computer display, respectively.

The operations of the lens distortion photographing and rotating face board photographing to determine the motion blur were described in “Quantifying Taxicab security camera Performance”.

Three pictures were taken in each of the defined conditions, resulting: in 3564 facial photographs with varying blurriness, 2970 overexposed facial photographs, 2970 underexposed facial photographs, and 1080 motion blurred facial photographs taken by the reference cameras. A total of 900 lens-distorted facial photographs were altered by using Photoshop CS5 software. In total, 11,484 valid photographs were taken in various camera setup, light, and seat conditions.

#### 5) Photograph evaluation by photograph evaluators

From the total number of valid photographs, 4428 facial photographs were selected and categorized by photographic-image quality parameters and light and seat conditions. The sorted photograph files were sent to 13 volunteer photograph evaluators for threshold votes. All of these photograph evaluators had experience in in-cab customer face identifications. Nine of them even had court experience in in-cab customer face identifications. The photograph evaluators examined the facial photographs and voted for the thresholds of five photographic-image quality parameters in each of the four in-cab light conditions and three seat positions (MB thresholds in rear-middle seat). For each of the photographic-image quality parameters, a photograph evaluator had 10 votes since there was a group of 10 subject facial photograph for each parameter. Thirteen evaluators had 130 votes for each of 78 parameter thresholds (18 thresholds for each of RES, DRS, DRH and LD, and 6 thresholds for MB).

#### 6) Statistical analysis of evaluator votes

Non-parametric approaches were used for this study. For each light condition and seat position, the median was computed from 10 votes by each of 13 photograph evaluators. The calculated 13 medians were then used to calculate the median and their associated distribution-free 95% confidence limits [34]. All of the analyses for this paper were generated using SAS/BASE software, Version 9.2 of the SAS System [35].

## 3. Results

### 3.1. Determination of Human Head-Shaking Speed

The mean angular speed of human head shaking was determined as 18.7 rpm. During MB threshold determination, the rotating face board rotated at this angular speed to simulate common human head-shaking speed.

### 3.2. Open Field Light Condition Measurements and In-Cab Light Condition Simulation

The in-cab light intensity and light color temperature were measured in the simulated taxicab in the open field in real L1 (daylight), L2 (moonless dark), L3 (moonless dark with backlight), and L4 (sunset via rear window) light conditions. The brightest light intensity is L1 condition outside the taxicab (115,320 lux), which is typical daylight intensity in the sun [30]. In each of the three seats, the light meter measured the light intensity in four directions (forward, left, right, and backward). The light intensities and color temperatures in three cab seats are shown in Table 1.

The light intensities and colors in the front-left and rear-left seats were omitted due to the seat symmetry inside a taxicab. The intensity data show that the light intensity in cab seats is orientation dependent. The light intensity difference in different directions could be as large as 19 times (such as L4 in rear-middle seat). In order to simulate the open field light conditions as accurately as possible during the light reconstruction in the simulated taxicab, the light intensities in four directions in each seat listed in Table 1 were reconstructed by using seven LED light panels and four incandescent light bulbs surrounding the simulated taxicab. The reconstructed light intensities in each of four directions of each of three seats in each of four light conditions were maintained within 10% of the light intensities listed in Table 1 during the reference camera tests.

### 3.3. Threshold Determination for Photograph Resolution (RES)

In photograph resolution threshold determination, the photograph test chart with the slanted square inset and 10 subject face insets were photographed one-by-one by the color reference camera and black-and-white reference camera with infrared capability (infrared camera) in four light conditions (L1 and L4 for color camera and L1, L2, L3, and L4 for infrared camera) and three seat conditions (front-right, rear-right, and rear-middle). Table 2 shows the photograph resolution thresholds and the associated 95% confidence intervals. The numbers of the votes by each of thirteen photograph evaluators are also shown in Table 2.

**Figure 11:**
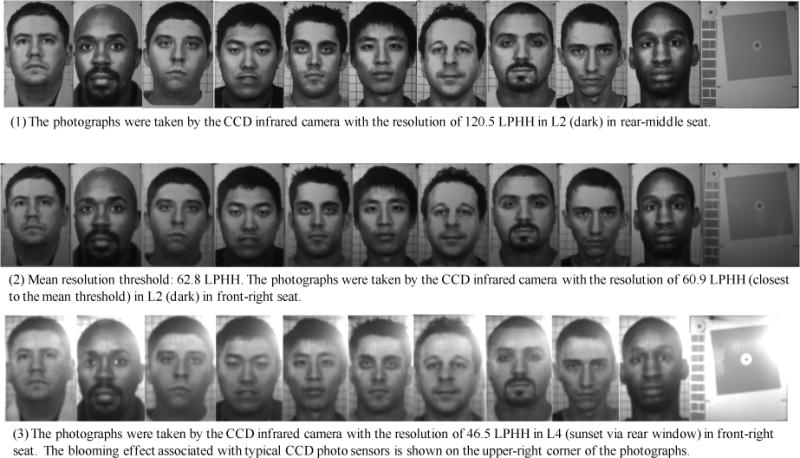
Photograph resolution thresholds: (1) highest, 120.5 LPHH; (2) mean, 62.8 LPHH; and (3) lowest, 46.5 LPHH.

The photograph evaluators voted for 18 photograph resolution thresholds in four light and three seat combinations by two reference cameras. These resolution thresholds ranged between 46.5 and 120.5 LPHH. Among these RES thresholds the highest (worst-case scenario) threshold is 120.5 LPHH in L2 (dark) in the rear-middle seat position. The lowest RES threshold is 46.5 LPHH in L4 (sunset) in the front-right seat position. The mean RES threshold of all of 18 thresholds is 62.8 LPHH. Figure 11 shows the face photographs voted by the photograph evaluators to be the highest and lowest RES thresholds.

**Figure 12:**
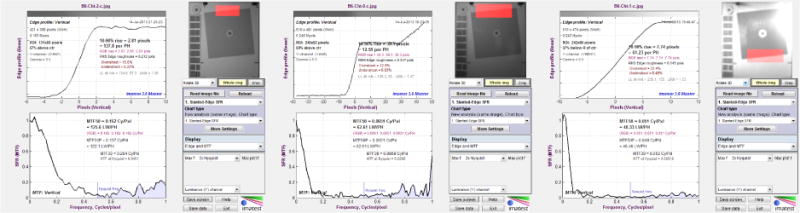
The modulation transfer function (MTF) measurement charts (using Imatest software) for the following photograph resolution measurements: (1) 122.1 LPHH; (2) 62.61 LPHH; and (3) 46.46 LPHH.

The face photographs, with their resolution close to the mean resolution threshold, are also shown in Figure 11. The slanted-squares are shown in Figure 12 after the corresponding facial images. Figure 12 shows the charts of the resolution measurements on the slanted squares using Imatest software. Imatest software measured the resolution of the slanted squares by computing the modulation transfer function (MTF) of the squares versus the spatial frequencies.

### 3.4. Threshold Determination for Photograph Highlight Dynamic Range (DRH)

In dynamic range threshold determination, the photograph-test charts with the 10 subject face insets were photographed one-by-one by the color and infrared reference cameras in the same light and seat conditions as in resolution threshold determination. Table 3 shows the 18 voted highlight dynamic range thresholds with each of light/seat condition combinations.

**Figure 13:**
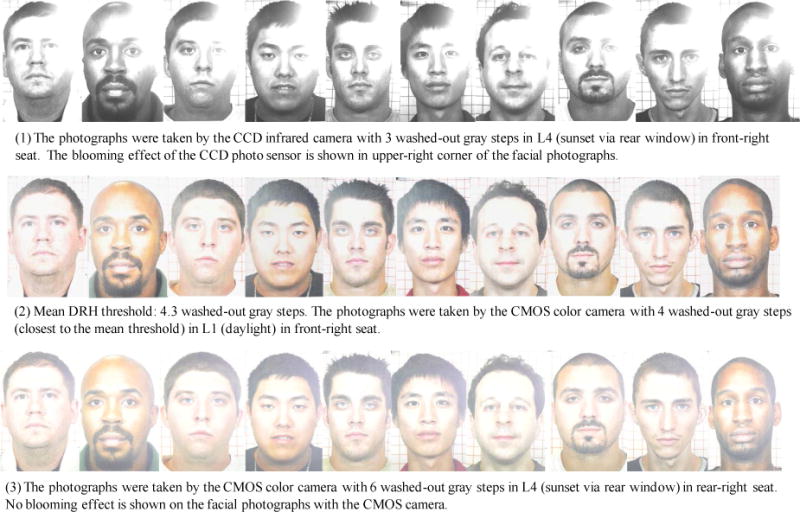
Photograph highlight-dynamic-range thresholds: (1) highest is 3 washed-out gray steps, (2) mean is 4.3 washed-out gray steps, and (3) lowest is 6 washed-out gray steps.

The thresholds are distributed in a range between three and six washed-out gray steps. In the worst-case scenario, the highest DRH threshold is no more than three washed-out gray steps (1 EV), and the lowest DRH threshold is no more than six washed-out gray steps (2 EV). The mean DRH threshold is no more than 4.3 washed-out gray steps (1.4 EV). Figure 13 shows the facial photographs voted by the photograph evaluators to be the highest and lowest DRH thresholds. The facial photographs, with their DRH close to the mean DRH threshold, are also shown in Figure 13.

### 3.5. Threshold Determination for Photograph Shadow Dynamic Range (DRS)

Table 4 shows the 18 voted shadow dynamic range thresholds with each of light/seat condition combinations. The thresholds are distributed in a range between 7 and 10 recognizable gray steps.

**Figure 14:**
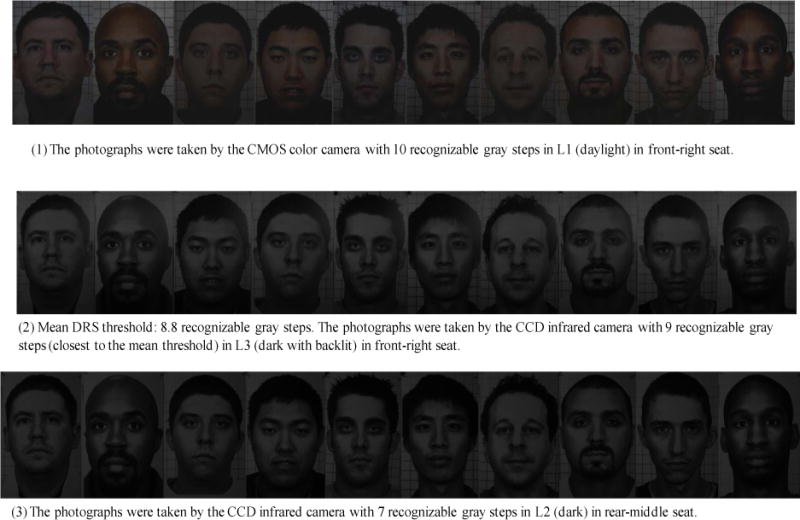
Photograph shadow-dynamic-range thresholds: (1) highest is 10 recognizable gray steps, (2) mean is 8.8 recognizable gray steps, and (3) lowest is 7 recognizable gray steps.

In the worst-case scenario, the highest DRS threshold is at least 10 recognizable gray steps (3.3 EV), and the lowest DRS threshold is at least 7 recognizable gray steps (2.3 EV). The mean DRS threshold is at least 8.8 recognizable gray steps (2.9 EV). Figure 14 shows the facial photographs voted by the photograph evaluators to be the highest and lowest DRS thresholds. The facial photographs, with their DRS close to the mean DRS threshold, are also shown in Figure 14.

### 3.6. Threshold Determination for Photograph Lens Distortion (LD)

In lens distortion threshold determination, the photograph-test charts with 10 gridded facial image insets were photographed one-by-one by the color and infrared reference cameras in the same light and seat conditions as in resolution threshold determination. Table 5 shows the lens distortion thresholds for 18 light/seat condition combinations. The LD thresholds are distributed in a range between 30% and 60%.

**Figure 15:**
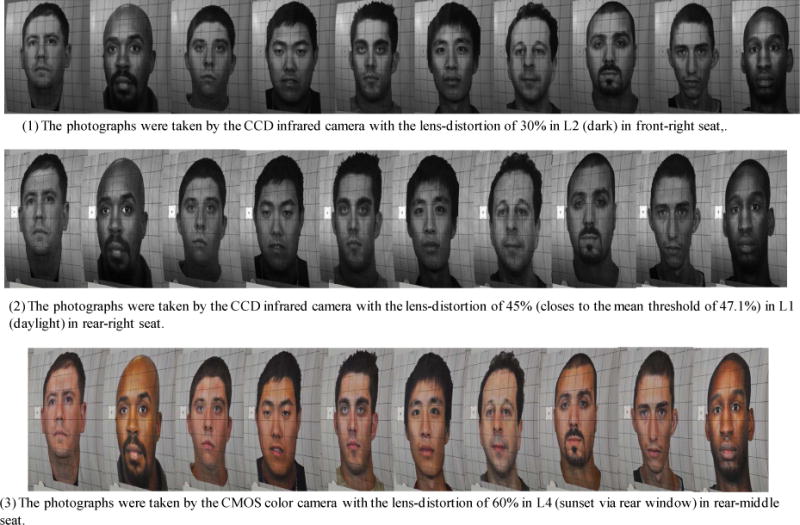
Photograph lens-distortion thresholds: (1) highest, 30%; (2) mean, 47.1%; and (3) lowest, 60%.

In the worst-case scenario, the highest LD threshold is no more than 30%, and the lowest LD threshold is no more than 60%. The mean LD threshold is no more than 47.1%. Figure 15 shows the facial photographs voted by the photograph evaluators to be the highest and lowest LD thresholds. The facial photographs, with their lens distortion close to the mean LD threshold, are also shown in Figure 15.

### 3.7. Threshold Determination for Photograph Motion Blur (MB)

In motion blur threshold determination, the rotating face board, covered with 10 facial pages (one at a time), was spinning at a constant angular speed of 18.73 rpm. This angular speed represents common human head-movement speed, which was determined in human subject head-shaking experiments. Table 6 shows the motion blur shutter speed thresholds in six light/camera conditions in the rear-middle seat. The MB thresholds are distributed in a narrow range between 33.3 mS and 36.7 mS.

**Figure 16:**
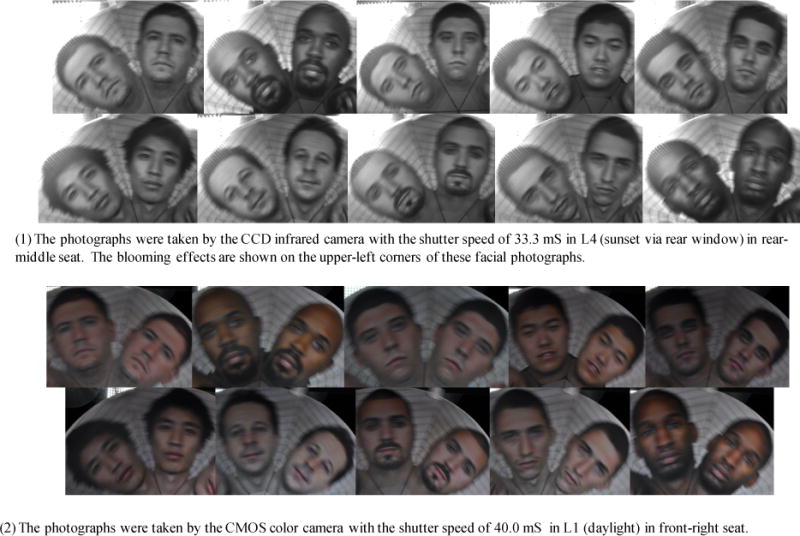
Motion-blur facial images with their blurriness (1) equal to the highest threshold (33.3 mS) and (2) close to the lowest threshold (36.65 mS).

In the worst-case scenario, the highest MB threshold is no more than 33.3 mS, and the lowest MB threshold is no more than 36.7 mS. The mean MB threshold is no more than 33.9 mS. Figure 16 shows two groups of facial images photographed with the shutter speeds equal to 33.3 mS and close to 36.7 mS. The captured rotating facial photographs became more blurry as the shutter speed became slower.

### 3.8. Summary of 78 Camera Parameter Thresholds

A summary of 78 taxicab security camera parameter thresholds is shown in Table 7. Table 7 shows the median thresholds with the 95% confidence levels and their lower/upper boundaries of the five photographic metrics under each of the combined test conditions.

## 4. Discussion

### 4.1. Photograph Resolution Thresholds

The 18 resolution (RES) thresholds in different lights and seats are within the range of 46.5 lines per head height (LPHH) and 120.5 LPHH. In the reference camera tests, the different facial image resolutions were obtained during photographing by manually adjusting the lens focal length. Obtaining uniformed resolution distributions was difficult using this method. The resolutions of the facial photographs taken by the CCD infrared camera in L2 (dark) conditions in rear-middle seat were not uniformly distributed. The RES interval between the third RES level (133.2 LPHH) and the fourth RES level (120.5 LPHH) is 12.7 LPHH, which is much smaller than that between the fourth RES level (120.5 LPHH) and the fifth RES level (51.7 LPHH), which is 68.8 LPHH. Since all of the evaluator votes were concentrated on 120.5 LPHH (69 votes) and 51.7 LPHH (61 votes) in this light/seat condition, a reasonable RES threshold in this condition should be the mean of these two RES values: 88.2 LPHH, which is the worst-case scenario among 18 RES thresholds. This resolution was measured using a simulated head height of 30 cm. A 99^th^ percentile male head height was determined as 25.5 cm by Human Engineering Design Data Digest [36]. Therefore, the worst-case scenario resolution for a 99^th^ percentile male head should be 88.2/30 × 25.5 = 74.97 LPHH. The worst-case scenario resolution of 75 LPHH is suggested as the minimum requirement for photographic image resolution.

The minimum RES threshold of 75 LPHH is a serious challenge to cameras equipped with VGA photographic image sensors, which are commonly equipped in taxicab security camera systems. The pixel count of a VGA sensor is 640 (horizontal) × 480 (vertical) pixels. The horizontal field of view of a taxicab security camera lens should be wide enough to include customers in both of the front- and back-seats. The vertical field of view should cover both the front and back seat customers from head to hips. For the purpose of customer facial identification, the field of view of the camera should also be limited so that a customer’s face is large enough to be identified. The Australian closed-circuit TV guidelines specified that a customer’s head height should be no less than 15% of the picture height for face identification [37]. As a result, in a captured in-cab photograph the head height of a rear-seat customer is no less than 15% of the picture height of the photograph. In order to maintain the 75 LPHH of minimum facial resolution, the vertical resolution of an image sensor should be no less than 500 line-widths. The sensor vertical pixel count should be further increased as the resolution loss caused by sensor noise, Bayer demosaicing [38], lossy image compression, and lens resolution are accounted for in resolution reduction. The actual resolution loss varies with different individual camera design, for example, the actual resolution of a comparable point-and-shoot camera is about two thirds of its image sensor pixel count [39]. Therefore, the taxicab security camera sensor should have 75/0.15 × 3/2 = 750 vertical pixels. A VGA image sensor with 480 vertical pixels cannot satisfy the minimum RES requirement. Therefore, to meet the minimum RES requirement, a taxicab security camera system should be equipped with an XGA format image sensor (1024 horizontal × 768 vertical pixels) or with a higher image resolution format.

### 4.2. Dynamic Range Thresholds

**Figure 17:**
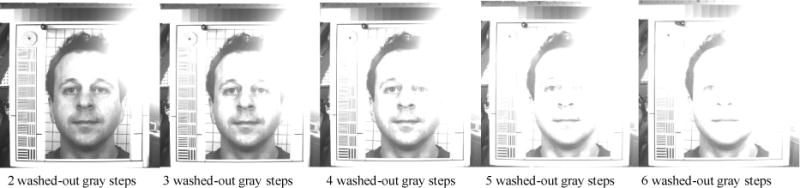
Blooming effect on facial photographs taken by the CCD camera is more vulnerable with more washed-out gray steps in the 14-inch Kodak Q-14 Gray Scale Chart above the facial chart.

A taxicab security camera without enough highlight dynamic range could take washed-out in-cab face photographs in some extreme taxicab light conditions, such as L3 (dark with backlight) and L4 (sunset via rear window). Taxicab security cameras equipped with CCD image sensors could deteriorate the washed-out photographing scenario. Figures 13 (1) and Figures 16 (1) show some partially washed-out photographs taken by the reference camera with a CCD image sensor in L4. Figure 17 shows a series of partially washed-out facial photographs taken by the same CCD camera in L4 with two to six washed-out gray steps in the Kodak Gray Scale Chart above the human subject face chart. These washed-out photographs were caused by the backlight.

The washed-out image area becomes larger with more washed-out gray steps. The washed-out phenomenon is more severe with CCD image sensors than with CMOS image sensors, attributed to CCD sensor’s blooming effect characteristics. A CCD sensor overflows excessive electrical charges from overexposed pixels to the neighborhood pixels, which enlarges the washed-out image area [40]. To avoid the washed-out photographs in strong backlights, the blooming effect caused by CCD sensors should be considered in taxicab security camera design. One camera design option is to substitute CCD sensors with CMOS sensors. High dynamic range (HDR) imaging is another option to be applied to taxicab security camera design to mitigate the washed-out effect [41]. By sequentially taking multiple pictures with different exposure and combining these pictures into one frame of image in HDR imaging, the lost image information in the washed-out picture area might be compensated by low-exposure pictures.

In order to measure the photographic image resolution, there was a 30 × 27.5 cm rectangular outline and the light-gray background surrounding a facial image inset on the photograph-test chart (Figure 3(b)). These outlines and backgrounds blocked the backlights from the rear window. This backlight blockage mitigated the vulnerability of the blooming effect on facial images taken by the CCD camera in L3 (dark with backlight) and L4 (sunset via rear window) conditions. The test photographic images show that it would take two more washed-out gray steps of overexposure (0.7 EV) to expand the blooming washed-out image area from the chart edge to reach the facial image. Therefore, two gray-steps of compensation (0.7 EV) were subtracted from the minimum highlight dynamic range requirement for CCD cameras. The backlight blockage did not cause blooming washed-out effect on the facial image insets taken by the CMOS camera.

In order to improve the photographic shadow dynamic range of a taxicab security camera, it is important that a well and uniformly lit taxicab be maintained using infrared LEDs in L2 (dark), L3 (dark with backlight), and even L4 (sunset via rear window) light conditions. In order to protect the taxicab customer eyes, the infrared LED light intensity should be kept below the maximum permissible exposure for human eyes to a laser beam specified in American National Standard for Safe Use of Lasers [32].

The worst-case scenario DRH threshold of no more than three washed-out gray steps (1.0 EV) is suggested as the minimum photographic highlight dynamic range requirement for CMOS cameras. For CCD cameras with blooming effect, this requirement should be no more than one washed-out gray step (0.3 EV).

The worst-case scenario DRS threshold of at least 10 recognizable gray steps (3.3 EV) is suggested as the minimum photographic shadow dynamic range requirement. The unit of the gray steps in Kodak Gray Scale Chart was used to measure the highlight and shadow dynamic ranges in reference camera dynamic range tests in order to easily explain the physical meaning of the test results. The gray steps can be easily converted to exposure values (EV), which is the unit commonly used in making camera settings. One gray step equals 1/3 EV.

### 4.3. Lens Distortion Thresholds

Among 18 lens distortion-related thresholds voted by 13 photograph evaluators, the LD threshold range spreads from 30% to 60%. The explanation of the wide spread of lens distortion threshold might be that the human eyes are not sensitive to lens distortion in facial identification. The worst-case scenario LD threshold of no more than 30% is suggested as the minimum lens distortion requirement. This minimum requirement is not a serious challenge for a common taxicab security camera since a taxicab security camera’s lens distortion would rarely reach 30%.

### 4.4. Motion Blur Threshold

The photograph evaluators voted on six MB thresholds in six light/camera conditions in the rear-middle seat. The worst-case scenario MB threshold of no more than 1/30 second is suggested as the minimum shutter speed requirement for motion blur control. These MB thresholds do not seem to be a serious challenge for common taxicab security cameras since most of them are able to take taxicab photographs with shutter speeds at or above these MB thresholds.

### 4.5. Future Research

1. Since there are many different vehicle types and layouts that are being used as taxicabs (small sedans, small hybrid vehicles, large sedans, SUVs, minivans, full size vans, etc.), there are different sets of video configurations. These configurations relate to the actual seat locations and their distance from camera mounting, and the lighting considerations for a proper capture in each of those vehicles and layouts. Similar camera tests should be conducted in the future to determine minimum technical requirements for taxicab security cameras used in these vehicles.
2. In some small hybrid taxicabs, due to the windshield configuration the camera must be mounted off center. Thus, cameras with wider viewing angles are needed for off-center mounting. More camera tests should be conducted in the future to determine the minimum requirement for the viewing angle of taxicab security cameras.
3. There are 18 thresholds under different lighting conditions and seat positions for each of four photographic metrics (resolution, highlight dynamic range, shadow dynamic range and lens distortion) and 6 thresholds for motion blur photographic metric. Data reduction should be considered in the future research to reduce the number of thresholds by combining latent variables through factor analysis or other methods.
4. The use of HDR imaging is discussed in this paper to increase the photographic dynamic range of in-taxicab facial images. The legal status of HDR imaging in court facial identification should be explored in the future.
5. All of the facial images used in facial identification in this research were front-face images. The off-axis facial identification should be explored in the future research. Other factors, such as an individual wearing a cap, a hood or glasses, should be considered in the future in-taxicab facial identification research.
6. The human subjects in this taxicab security camera test study were all male. Some taxicab security camera manufacturers had been helping police download video clips from taxi incidents and many of them involved female passengers. It is reasonable that female human subjects should also be recruited in the future research.
7. Since the rear-left seat is the first choice for many criminals or malevolent individuals, the rear-left seat should be considered as one of the preferred seat position for the future taxicab security camera tests.
8. In-taxicab facial recognition by facial recognition software should also be explored in the future for potential to reduce subjective identification errors in manual facial identification.

## 5. Conclusions

Seventy-eight minimum-image quality thresholds for an effective taxicab security camera system were voted on by 13 photograph evaluators with taxicab facial identification experience. A taxicab security camera system that operates with these thresholds should be able to record identifiable customer face images inside a taxicab in various light conditions and seat positions. Among these thresholds, this research suggests five worst-case scenario thresholds as the minimum taxicab security camera parameter requirements. The image resolution threshold is at least 75 LPHH for a 99^th^ percentile human head. The highlight dynamic range threshold is no more than 1 EV washed-out gray steps for CMOS cameras and no more than 0.3 EV washed-out gray steps for CCD cameras with blooming effects. The shadow dynamic range threshold is at least 3.3 EV recognizable gray steps. The lens distortion threshold is no more than 30%, and the motion blur shutter speed threshold is no more than 1/30 second.

The resolution pixel count of a taxicab security camera image sensor should be equal to or higher than XGA image format (1024 × 768 pixels). Some factors should be considered by taxicab security camera manufacturers in camera design including: 1) maintaining a well and uniformly illuminated cab with an infrared light source in L2 (dark), L3 (dark with backlight), and L4 (sunset via rear window) light conditions; 2) the blooming effect of the CCD photograph sensors, which might deteriorate the facial image identification; and 3) the application of high-dynamic-range imaging to camera design to mitigate washed-out effect in backlight photographing.

## Figures and Tables

**Table 1:** Real light conditions measured in the open filed (mean of 5 measurements).

Light Conditions		L1-Day Light	L2-Dark	L3-Dark + Backlit	L4-Sunset via Rear Window
**Outside Taxicab**	Sky-Lumin. (lux)	115320.00	0.52	1.76	3120.00
	Sun-Lumin. (lux)	113080.00	X	423.20	9304.00
	Sky Color Temp.(°k)	5524.00	X	X	6156.00
	Sun Color Temp. (°k)	4242.88	X	3158.00	3264.00

**Front-Right Seat**	Left Window (lux)	1840.00	0.14	7.88	666.20
	Right Window (lux)	7412.00	0.40	6.38	1702.00
	Windshield (lux)	4172.00	0.10	1.14	725.40
	Rear Window (lux)	1763.60	0.22	76.90	4088.00
	Light Color (°k)	6450.00	X	X	4966.00

**Rear-Right Seat**	Left Window (lux)	1710.60	0.12	18.73	1623.20
	Right Window (lux)	5152.00	0.13	11.33	2418.00
	Windshield (lux)	1641.60	0.05	0.71	587.40
	Rear Window (lux)	2632.00	0.22	90.72	6314.00
	Light Color (°k)	6536.00	X	X	4660.00

**Rear-Middle Seat**	Left Window (lux)	2348.00	0.09	12.74	1546.60
	Right Window (lux)	2696.00	0.09	18.65	2188.00
	Windshield (lux)	1495.00	0.05	0.81	425.20
	Rear Window (lux)	3250.00	0.30	96.66	8144.00
	Light Color (°k)	6444.00	X	X	5182.00

**Table 2:** Taxicab facial photo evaluation study: resolution votes. Unit: Lines per Head Height (LPPH) (Head-Height = 30 cm).

Seat Positions		Front-Right-Seat						Rear-Right-Seat						Rear-Middle-Seat					

Resolution: LPPH		953.8	129.4	101.5	75.2	56.2	31.0	900.9	203.7	94.7	83.2	56.1	22.6	897.8	192.7	168.7	72.7	54.3	26.8
**Daylight (L1)/NK Camera**	**Total Votes**	**0**	**0**	**7**	**40**	**79**	**4**	**0**	**0**	**9**	**54**	**65**	**2**	**0**	**1**	**39**	**53**	**35**	**2**
	**Evaluator 1**	0	0	0	0	10	0	0	0	0	0	10	0	0	0	0	0	10	0
	**2**	0	0	0	1	9	0	0	0	5	5	0	0	0	0	10	0	0	0
	**3**	0	0	0	6	4	0	0	0	0	1	9	0	0	0	7	3	0	0
	**4**	0	0	0	0	10	0	0	0	0	10	0	0	0	0	0	10	0	0
	**5**	0	0	0	6	4	0	0	0	0	1	9	0	0	0	7	3	0	0
	**6**	0	0	0	5	5	0	0	0	0	1	9	0	0	0	7	3	0	0
	**7**	0	0	0	0	8	2	0	0	0	0	10	0	0	0	0	0	10	0
	**8**	0	0	0	0	8	2	0	0	0	0	10	0	0	0	0	0	10	0
	**9**	0	0	2	5	3	0	0	0	3	7	0	0	0	1	7	2	0	0
	**10**	0	0	0	7	3	0	0	0	0	10	0	0	0	0	1	9	0	0
	**11**	0	0	0	2	8	0	0	0	1	9	0	0	0	0	0	10	0	0
	**12**	0	0	5	5	0	0	0	0	0	10	0	0	0	0	0	10	0	0
	**13**	0	0	0	3	7	0	0	0	0	0	8	2	0	0	0	3	5	2
	**Median**	**56.2**						**56.1**						**72.7**					
	**95% CI**	**Lower**	56.2		**Upper**	75.2		**Lower**	56.1		**Upper**	83.2		**Lower**	54.3		**Upper**	169	

Seat Positions		Front-Right-Seat						Rear-Right-Seat						Rear-Middle-Seat					

Resolution (LPPH)		516.4	133.4	111.0	61.7	59.9	28.9	399.8	181.8	141.8	110.1	55.4	25.2	347.0	109.2	93.7	69.6	58.5	25.1
**Daylight (L1)/PG Camera**	**Total Votes**	**0**	**0**	**15**	**79**	**27**	**9**	**0**	**0**	**0**	**73**	**56**	**1**	**0**	**3**	**22**	**44**	**60**	**1**
	**Evaluator 1**	0	0	0	4	6	0	0	0	0	0	10	0	0	0	0	0	10	0
	**2**	0	0	0	10	0	0	0	0	0	10	0	0	0	0	8	2	0	0
	**3**	0	0	1	9	0	0	0	0	0	4	6	0	0	0	0	6	4	0
	**4**	0	0	0	10	0	0	0	0	0	10	0	0	0	0	0	10	0	0
	**5**	0	0	1	9	0	0	0	0	0	4	6	0	0	0	0	6	4	0
	**6**	0	0	1	9	0	0	0	0	0	4	6	0	0	0	0	6	4	0
	**7**	0	0	0	10	0	0	0	0	0	0	10	0	0	0	0	0	10	0
	**8**	0	0	0	10	0	0	0	0	0	0	10	0	0	0	0	0	10	0
	**9**	0	0	0	7	3	0	0	0	0	8	2	0	0	3	7	0	0	0
	**10**	0	0	4	0	6	0	0	0	0	10	0	0	0	0	0	7	3	0
	**11**	0	0	0	0	10	0	0	0	0	10	0	0	0	0	7	3	0	0
	**12**	0	0	8	0	2	0	0	0	0	10	0	0	0	0	0	3	7	0
	**13**	0	0	0	1	0	9	0	0	0	3	6	1	0	0	0	1	8	1
	**Median**	**61.7**						**55.4**						**69.6**					
	**95% CI**	**Lower**	59.9		**Upper**	61.7		**Lower**	55.4		**Upper**	110		**Lower**	58.5		**Upper**	93.7	

Seat Positions		Front-Right-Seat						Rear-Right-Seat						Rear-Middle-Seat					

Resolution (LPPH)		234.8	143.0	102.5	60.9	52.4	21.5	355.5	119.2	106.6	93.6	55.0	20.7	188.7	150.2	133.2	120.5	51.7	25.0
**Dark (L2)/PG Camera**	**Total Votes**	**0**	**0**	**18**	**64**	**47**	**1**	**0**	**0**	**2**	**54**	**71**	**3**	**0**	**0**	**0**	**69**	**61**	**0**
	**Evaluator 1**	0	0	0	0	10	0	0	0	0	0	10	0	0	0	0	10	0	0
	**2**	0	0	5	3	2	0	0	0	0	10	0	0	0	0	0	10	0	0
	**3**	0	0	1	7	2	0	0	0	0	0	10	0	0	0	0	0	10	0
	**4**	0	0	0	10	0	0	0	0	0	10	0	0	0	0	0	10	0	0
	**5**	0	0	1	6	2	1	0	0	0	0	10	0	0	0	0	0	10	0
	**6**	0	0	1	5	4	0	0	0	0	0	10	0	0	0	0	0	10	0
	**7**	0	0	0	0	10	0	0	0	0	0	10	0	0	0	0	0	10	0
	**8**	0	0	0	1	9	0	0	0	0	0	10	0	0	0	0	0	10	0
	**9**	0	0	0	5	5	0	0	0	0	6	4	0	0	0	0	10	0	0
	**10**	0	0	0	10	0	0	0	0	0	10	0	0	0	0	0	10	0	0
	**11**	0	0	4	6	0	0	0	0	2	8	0	0	0	0	0	10	0	0
	**12**	0	0	3	7	0	0	0	0	0	9	1	0	0	0	0	8	2	0
	**13**	0	0	3	4	3	0	0	0	0	1	6	3	0	0	0	1	9	0
	**Median**	**60.9**						**55**						**120.5**					
	**95% CI**	**Lower**	52.4		**Upper**	60.9		**Lower**	55		**Upper**	93.6		**Lower**	51.7		**Upper**	121	

Seat Positions		Front-Right-Seat						Rear-Right-Seat						Rear-Middle-Seat					

Resolution (LPPH)		176.5	121.0	105.4	89.9	55.9	25.8	178.3	121.9	96.6	71.5	49.6	25.6	161.3	154.8	118.2	96.9	47.4	23.5
**Dark-BL (L3)/PG Camera**	**Total Votes**	**0**	**0**	**0**	**65**	**65**	**0**	**0**	**0**	**1**	**57**	**71**	**1**	**0**	**0**	**7**	**42**	**81**	**0**
	**Evaluator 1**	0	0	0	0	10	0	0	0	0	0	10	0	0	0	0	0	10	0
	**2**	0	0	0	0	10	0	0	0	0	10	0	0	0	0	0	10	0	0
	**3**	0	0	0	8	2	0	0	0	0	0	10	0	0	0	0	0	10	0
	**4**	0	0	0	10	0	0	0	0	0	10	0	0	0	0	0	0	10	0
	**5**	0	0	0	9	1	0	0	0	0	0	10	0	0	0	0	0	10	0
	**6**	0	0	0	9	1	0	0	0	0	0	10	0	0	0	0	0	10	0
	**7**	0	0	0	5	5	0	0	0	0	0	10	0	0	0	0	0	10	0
	**8**	0	0	0	0	10	0	0	0	0	0	10	0	0	0	0	0	10	0
	**9**	0	0	0	7	3	0	0	0	0	6	4	0	0	0	7	3	0	0
	**10**	0	0	0	2	8	0	0	0	0	10	0	0	0	0	0	10	0	0
	**11**	0	0	0	1	9	0	0	0	1	9	0	0	0	0	0	10	0	0
	**12**	0	0	0	10	0	0	0	0	0	10	0	0	0	0	0	9	1	0
	**13**	0	0	0	4	6	0	0	0	0	2	7	1	0	0	0	0	10	0
	**Median**	**72.9**						**49.6**						**47.4**					
	**95% CI**	**Lower**	55.9		**Upper**	89.9		**Lower**	49.6		**Upper**	71.5		**Lower**	47.4		**Upper**	96.9	

Seat Positions		Front-Right-Seat						Rear-Right-Seat						Rear-Middle-Seat					

Resolution (LPPH)		1005.7	124.5	101.1	72.8	57.2	31.3	927.0	123.1	95.2	53.8	48.4	21.6	989.5	199.4	163.6	118.8	52.8	21.0
**Sunset-RW (L4)/NK Camera**	**Total Votes**	**0**	**0**	**13**	**39**	**76**	**2**	**0**	**0**	**56**	**58**	**14**	**2**	**0**	**0**	**0**	**46**	**83**	**1**
	**Evaluator 1**	0	0	0	0	10	0	0	0	0	10	0	0	0	0	0	0	10	0
	**2**	0	0	0	0	10	0	0	0	10	0	0	0	0	0	0	10	0	0
	**3**	0	0	0	4	6	0	0	0	0	6	4	0	0	0	0	0	10	0
	**4**	0	0	0	10	0	0	0	0	10	0	0	0	0	0	0	0	10	0
	**5**	0	0	0	4	6	0	0	0	0	6	4	0	0	0	0	0	10	0
	**6**	0	0	0	5	5	0	0	0	0	6	4	0	0	0	0	0	10	0
	**7**	0	0	0	4	6	0	0	0	0	10	0	0	0	0	0	0	10	0
	**8**	0	0	0	0	10	0	0	0	0	10	0	0	0	0	0	0	10	0
	**9**	0	0	2	6	2	0	0	0	6	3	1	0	0	0	0	10	0	0
	**10**	0	0	1	5	4	0	0	0	10	0	0	0	0	0	0	10	0	0
	**11**	0	0	0	1	9	0	0	0	10	0	0	0	0	0	0	10	0	0
	**12**	0	0	10	0	0	0	0	0	10	0	0	0	0	0	0	6	4	0
	**13**	0	0	0	0	8	2	0	0	0	7	1	2	0	0	0	0	9	1
	**Median**	**57.2**						**53.8**						**52.8**					
	**95% CI**	**Lower**	57.2		**Upper**	72.8		**Lower**	53.8		**Upper**	95.2		**Lower**	52.8		**Upper**	119	

Seat Positions		Front-Right-Seat						Rear-Right-Seat						Rear-Middle-Seat					

Resolution (LPPH)		475.1	222.3	93.6	46.5	36.9	35.4	364.8	150.9	108.5	87.1	55.7	29.0	355.6	164.7	152.5	126.1	55.6	28.1
**Sunset-RW (L4)/PG Camera**	**Total Votes**	**0**	**0**	**46**	**68**	**7**	**9**	**0**	**0**	**41**	**29**	**58**	**2**	**0**	**1**	**0**	**48**	**79**	**2**
	**Evaluator 1**	0	0	0	10	0	0	0	0	10	0	0	0	0	0	0	0	10	0
	**2**	0	0	0	10	0	0	0	0	8	2	0	0	0	0	0	10	0	0
	**3**	0	0	0	10	0	0	0	0	0	0	10	0	0	0	0	0	10	0
	**4**	0	0	0	10	0	0	0	0	0	10	0	0	0	0	0	0	10	0
	**5**	0	0	0	10	0	0	0	0	0	0	10	0	0	0	0	0	10	0
	**6**	0	0	1	9	0	0	0	0	0	0	10	0	0	0	0	0	10	0
	**7**	0	0	9	1	0	0	0	0	0	0	10	0	0	0	0	0	10	0
	**8**	0	0	0	4	6	0	0	0	0	0	10	0	0	0	0	0	9	1
	**9**	0	0	7	3	0	0	0	0	8	2	0	0	0	1	0	9	0	0
	**10**	0	0	9	1	0	0	0	0	10	0	0	0	0	0	0	10	0	0
	**11**	0	0	10	0	0	0	0	0	3	7	0	0	0	0	0	10	0	0
	**12**	0	0	10	0	0	0	0	0	2	8	0	0	0	0	0	9	1	0
	**13**	0	0	0	0	1	9	0	0	0	0	8	2	0	0	0	0	9	1
	**Median**	**46.5**						**87.1**						**55.6**					
	**95% CI**	**Lower**	46.5		**Upper**	93.6		**Lower**	55.7		**Upper**	109		**Lower**	55.6		**Upper**	126	

**Mean of 18 Thresholds (LPHH)**							**62.8**			**Worst-Case Scenario Threshold (LPHH)**							88.2*		

* The Worst-Case Scenario Threshold = (120.5 × 69 + 51.7 × 61)/(69 + 61) = 88.2 LPHH (see discussion in Section 4.1)

**Table 3:** Taxicab facial photo evaluation study: Highlight Dynamic Range (DRH) Votes. Unit: Gray Steps (GS).

Seat Positions		Front-Right-Seat						Rear-Right-Seat						Rear-Middle-Seat					

DRH (Gray Step)		0.0	2.0	3.0	4.0	5.0	6.0	0.0	2.0	3.0	4.0	5.0	6.0	0.0	2.0	3.0	4.0	5.0	6.0
**Daylight (L1)/NK Camera**	**Total Votes**	**0**	**1**	**17**	**44**	**35**	**33**	**0**	**0**	**4**	**17**	**36**	**73**	**0**	**0**	**8**	**36**	**66**	**20**
	**Evaluator 1**	0	0	0	8	2	0	0	0	0	0	10	0	0	0	0	0	10	0
	**2**	0	0	6	3	1	0	0	0	0	6	4	0	0	0	0	8	2	0
	**3**	0	0	0	5	5	0	0	0	0	0	0	10	0	0	0	0	10	0
	**4**	0	0	0	0	5	5	0	0	0	0	0	10	0	0	0	8	2	0
	**5**	0	0	0	5	5	0	0	0	0	0	0	10	0	0	0	0	10	0
	**6**	0	0	0	6	4	0	0	0	0	0	0	10	0	0	0	0	10	0
	**7**	0	0	0	0	0	10	0	0	0	0	0	10	0	0	0	0	0	10
	**8**	0	0	0	0	0	10	0	0	0	0	0	10	0	0	0	2	7	1
	**9**	0	1	3	3	3	0	0	0	1	5	4	0	0	0	7	3	0	0
	**10**	0	0	0	1	1	8	0	0	0	0	2	8	0	0	0	3	5	2
	**11**	0	0	0	2	8	0	0	0	0	0	10	0	0	0	0	7	3	0
	**12**	0	0	4	5	1	0	0	0	3	6	1	0	0	0	1	4	5	0
	**13**	0	0	4	6	0	0	0	0	0	0	5	5	0	0	0	1	2	7
	**Median**	**4.5**						**6**						**5**					
	**95% CI**	**Lower**	4		**Upper**	6		**Lower**	4		**Upper**	6		**Lower**	4		**Upper**	5	

Seat Positions		Front-Right-Seat						Rear-Right-Seat						Rear-Middle-Seat					

DRH (Gray Step)		0.0	2.0	3.0	4.0	5.0	6.0	0.0	2.0	3.0	4.0	5.0	6.0	0.0	2.0	3.0	4.0	5.0	6.0
**Daylight (L1)/PG Camera**	**Total Votes**	**12**	**5**	**13**	**53**	**35**	**12**	**0**	**0**	**9**	**24**	**64**	**33**	**0**	**0**	**22**	**62**	**35**	**11**
	**Evaluator 1**	0	0	8	2	0	0	0	0	0	9	1	0	0	0	0	10	0	0
	**2**	0	0	0	5	5	0	0	0	9	1	0	0	0	0	10	0	0	0
	**3**	4	2	0	1	3	0	0	0	0	0	10	0	0	0	0	8	2	0
	**4**	0	0	0	10	0	0	0	0	0	0	10	0	0	0	0	10	0	0
	**5**	4	2	0	0	4	0	0	0	0	0	10	0	0	0	0	6	4	0
	**6**	4	1	1	0	4	0	0	0	0	0	10	0	0	0	0	6	4	0
	**7**	0	0	0	2	4	4	0	0	0	0	0	10	0	0	0	0	7	3
	**8**	0	0	0	0	3	7	0	0	0	0	0	10	0	0	0	0	6	4
	**9**	0	0	1	6	3	0	0	0	0	0	5	5	0	0	3	7	0	0
	**10**	0	0	0	9	1	0	0	0	0	1	7	2	0	0	0	4	5	1
	**11**	0	0	0	10	0	0	0	0	0	5	5	0	0	0	8	2	0	0
	**12**	0	0	3	7	0	0	0	0	0	8	1	1	0	0	1	8	1	0
	**13**	0	0	0	1	8	1	0	0	0	0	5	5	0	0	0	1	6	3
	**Median**	**4**						**5**						**4**					
	**95% CI**	**Lower**	2.5		**Upper**	5		**Lower**	4		**Upper**	5.5		**Lower**	4		**Upper**	5	

Seat Positions		Front-Right-Seat						Rear-Right-Seat						Rear-Middle-Seat					

DRH (Gray Step)		0.0	2.0	3.0	4.0	5.0	6.0	0.0	2.0	3.0	4.0	5.0	6.0	0.0	2.0	3.0	4.0	5.0	6.0
**Dark (L2)/PG Camera**	**Total Votes**	**0**	**4**	**28**	**40**	**51**	**7**	**0**	**21**	**60**	**36**	**12**	**1**	**0**	**11**	**43**	**73**	**3**	**0**
	**Evaluator 1**	0	0	0	4	5	1	0	9	1	0	0	0	0	10	0	0	0	0
	**2**	0	2	7	1	0	0	0	2	7	1	0	0	0	0	4	6	0	0
	**3**	0	0	0	6	4	0	0	0	6	4	0	0	0	0	0	10	0	0
	**4**	0	0	0	8	2	0	0	0	10	0	0	0	0	0	10	0	0	0
	**5**	0	0	0	2	8	0	0	0	6	4	0	0	0	0	0	10	0	0
	**6**	0	0	0	5	5	0	0	0	6	4	0	0	0	0	0	10	0	0
	**7**	0	0	0	2	6	2	0	0	0	4	6	0	0	0	0	9	1	0
	**8**	0	0	2	2	5	1	0	0	3	7	0	0	0	0	2	8	0	0
	**9**	0	0	1	4	5	0	0	0	3	7	0	0	0	0	6	4	0	0
	**10**	0	0	2	3	4	1	0	0	8	2	0	0	0	1	4	5	0	0
	**11**	0	0	8	2	0	0	0	5	5	0	0	0	0	0	10	0	0	0
	**12**	0	2	8	0	0	0	0	5	5	0	0	0	0	0	7	3	0	0
	**13**	0	0	0	1	7	2	0	0	0	3	6	1	0	0	0	8	2	0
	**Median**	**4.5**						**3**						**4**					
	**95% CI**	**Lower**	3		**Upper**	5		**Lower**	2.5		**Upper**	4		**Lower**	3		**Upper**	4	

Seat Positions		Front-Right-Seat						Rear-Right-Seat						Rear-Middle-Seat					

DRH (Gray Step)		0.0	2.0	3.0	4.0	5.0	6.0	0.0	2.0	3.0	4.0	5.0	6.0	0.0	2.0	3.0	4.0	5.0	6.0
**Dark-BL (L3)/PG Camera**	**Total Votes**	**0**	**0**	**38**	**58**	**18**	**16**	**0**	**29**	**44**	**51**	**6**	**0**	**0**	**1**	**59**	**33**	**37**	**0**
	**Evaluator 1**	0	0	9	1	0	0	0	10	0	0	0	0	0	0	10	0	0	0
	**2**	0	0	1	8	1	0	0	3	6	1	0	0	0	0	10	0	0	0
	**3**	0	0	0	9	1	0	0	0	4	6	0	0	0	0	0	6	4	0
	**4**	0	0	10	0	0	0	0	10	0	0	0	0	0	0	10	0	0	0
	**5**	0	0	0	9	1	0	0	0	4	6	0	0	0	0	0	6	4	0
	**6**	0	0	0	10	0	0	0	0	4	6	0	0	0	0	0	6	4	0
	**7**	0	0	0	8	2	0	0	0	1	8	1	0	0	0	0	2	8	0
	**8**	0	0	0	0	5	5	0	0	0	9	1	0	0	0	0	4	6	0
	**9**	0	0	5	5	0	0	0	0	4	6	0	0	0	0	8	2	0	0
	**10**	0	0	0	1	8	1	0	1	5	4	0	0	0	1	6	2	1	0
	**11**	0	0	3	7	0	0	0	4	6	0	0	0	0	0	8	2	0	0
	**12**	0	0	10	0	0	0	0	1	9	0	0	0	0	0	7	2	1	0
	**13**	0	0	0	0	0	10	0	0	1	5	4	0	0	0	0	1	9	0
	**Median**	**4**						**4**						**3**					
	**95% CI**	**Lower**	3		**Upper**	5		**Lower**	3		**Upper**	4		**Lower**	3		**Upper**	5	

Seat Positions		Front-Right-Seat						Rear-Right-Seat						Rear-Middle-Seat					

DRH (Gray Step)		0.0	2.0	3.0	4.0	5.0	6.0	0.0	2.0	3.0	4.0	5.0	6.0	0.0	2.0	3.0	4.0	5.0	6.0
**Sunset-RW (L4)/NK Camera**	**Total Votes**	**0**	**14**	**23**	**61**	**26**	**6**	**0**	**0**	**16**	**15**	**34**	**65**	**0**	**0**	**0**	**11**	**58**	**61**
	**Evaluator 1**	0	0	10	0	0	0	0	0	10	0	0	0	0	0	0	0	10	0
	**2**	0	0	0	4	6	0	0	0	0	5	5	0	0	0	0	0	10	0
	**3**	0	0	0	8	2	0	0	0	0	0	0	10	0	0	0	0	0	10
	**4**	0	0	0	10	0	0	0	0	0	0	10	0	0	0	0	0	10	0
	**5**	0	0	0	8	2	0	0	0	0	0	0	10	0	0	0	0	0	10
	**6**	0	0	0	9	1	0	0	0	0	0	0	10	0	0	0	0	0	10
	**7**	0	0	4	6	0	0	0	0	0	0	0	10	0	0	0	0	0	10
	**8**	0	0	0	0	5	5	0	0	0	0	0	10	0	0	0	0	1	9
	**9**	0	5	5	0	0	0	0	0	0	3	7	0	0	0	0	6	4	0
	**10**	0	0	2	7	1	0	0	0	0	1	4	5	0	0	0	5	5	0
	**11**	0	0	1	9	0	0	0	0	0	3	7	0	0	0	0	0	9	1
	**12**	0	9	1	0	0	0	0	0	6	3	1	0	0	0	0	0	9	1
	**13**	0	0	0	0	9	1	0	0	0	0	0	10	0	0	0	0	0	10
	**Median**	**4**						**5.5**						**5**					
	**95% CI**	**Lower**	3		**Upper**	5		**Lower**	4.5		**Upper**	6		**Lower**	5		**Upper**	6	

Seat Positions		Front-Right-Seat						Rear-Right-Seat						Rear-Middle-Seat					

DRH (Gray Step)		0.0	2.0	3.0	4.0	5.0	6.0	0.0	2.0	3.0	4.0	5.0	6.0	0.0	2.0	3.0	4.0	5.0	6.0
**Sunset-RW (L4)/PG Camera**	**Total Votes**	**0**	**39**	**54**	**28**	**9**	**0**	**0**	**37**	**25**	**42**	**11**	**15**	**0**	**0**	**0**	**48**	**69**	**13**
	**Evaluator 1**	0	10	0	0	0	0	0	10	0	0	0	0	0	0	0	10	0	0
	**2**	0	0	1	9	0	0	0	5	5	0	0	0	0	0	0	10	0	0
	**3**	0	6	4	0	0	0	0	0	0	10	0	0	0	0	0	0	8	2
	**4**	0	0	10	0	0	0	0	10	0	0	0	0	0	0	0	0	10	0
	**5**	0	6	4	0	0	0	0	0	0	10	0	0	0	0	0	0	8	2
	**6**	0	5	5	0	0	0	0	0	0	10	0	0	0	0	0	0	8	2
	**7**	0	1	6	3	0	0	0	0	0	0	0	10	0	0	0	0	8	2
	**8**	0	0	3	7	0	0	0	0	2	2	6	0	0	0	0	1	5	4
	**9**	0	6	4	0	0	0	0	1	7	2	0	0	0	0	0	6	4	0
	**10**	0	0	2	8	0	0	0	0	4	6	0	0	0	0	0	8	2	0
	**11**	0	3	7	0	0	0	0	10	0	0	0	0	0	0	0	10	0	0
	**12**	0	2	8	0	0	0	0	1	7	2	0	0	0	0	0	3	7	0
	**13**	0	0	0	1	9	0	0	0	0	0	5	5	0	0	0	0	9	1
	**Median**	**3**						**4**						**5**					
	**95% CI**	**Lower**	2		**Upper**	4		**Lower**	2		**Upper**	5		**Lower**	4		**Upper**	5	

**Mean of 18 Thresholds (Gray Step)**							**4.3**			**Worst-Case Scenario Threshold (Gray Step)**							**3**		

**Table 4:** Taxicab facial photo evaluation study: Shadow Dynamic Range (DRS) Votes. Unit: Gray Steps (GS).

Seat Positions		Front-Right-Seat						Rear-Right-Seat						Rear-Middle-Seat					

DRS (Gray Step)		20.0	10.0	9.0	8.0	7.0	6.0	20.0	10.0	9.0	8.0	7.0	6.0	20.0	10.0	9.0	8.0	7.0	6.0
**Daylight (L1)/NK Camera**	**Total Votes**	**7**	**63**	**36**	**23**	**1**	**0**	**19**	**23**	**31**	**47**	**9**	**1**	**24**	**52**	**29**	**24**	**1**	**0**
	**Evaluator 1**	7	3	0	0	0	0	10	0	0	0	0	0	0	10	0	0	0	0
	**2**	0	0	3	7	0	0	0	1	3	6	0	0	0	0	5	5	0	0
	**3**	0	9	1	0	0	0	0	0	2	8	0	0	0	10	0	0	0	0
	**4**	0	10	0	0	0	0	2	8	0	0	0	0	10	0	0	0	0	0
	**5**	0	9	1	0	0	0	0	0	2	8	0	0	0	10	0	0	0	0
	**6**	0	10	0	0	0	0	0	0	2	8	0	0	0	10	0	0	0	0
	**7**	0	3	4	3	0	0	0	0	2	3	5	0	0	0	4	6	0	0
	**8**	0	5	5	0	0	0	6	4	0	0	0	0	4	6	0	0	0	0
	**9**	0	5	5	0	0	0	0	8	2	0	0	0	10	0	0	0	0	0
	**10**	0	1	6	3	0	0	0	0	0	10	0	0	0	0	4	6	0	0
	**11**	0	0	6	4	0	0	0	2	8	0	0	0	0	2	8	0	0	0
	**12**	0	8	2	0	0	0	1	0	8	1	0	0	0	4	4	2	0	0
	**13**	0	0	3	6	1	0	0	0	2	3	4	1	0	0	4	5	1	0
	**Median**	**9.5**						**8**						**10**					
	**95% CI**	**Lower**	9		**Upper**	10		**Lower**	8		**Upper**	10		**Lower**	8		**Upper**	10	

Seat Positions		Front-Right-Seat						Rear-Right-Seat						Rear-Middle-Seat					

DRS (Gray Step)		13.0	10.0	9.0	8.0	7.0	6.0	13.0	10.0	9.0	8.0	7.0	6.0	12.0	10.0	9.0	8.0	7.0	6.0
**Daylight (L1)/PG Camera**	**Total Votes**	**29**	**56**	**25**	**10**	**7**	**3**	**0**	**2**	**30**	**60**	**16**	**22**	**9**	**47**	**17**	**15**	**42**	**0**
	**Evaluator 1**	2	8	0	0	0	0	0	1	9	0	0	0	0	10	0	0	0	0
	**2**	0	0	4	6	0	0	0	0	0	2	8	0	0	0	0	0	10	0
	**3**	8	2	0	0	0	0	0	0	0	10	0	0	0	10	0	0	0	0
	**4**	0	10	0	0	0	0	0	0	2	8	0	0	0	10	0	0	0	0
	**5**	9	1	0	0	0	0	0	0	0	10	0	0	0	8	2	0	0	0
	**6**	10	0	0	0	0	0	0	0	0	10	0	0	0	8	2	0	0	0
	**7**	0	6	2	2	0	0	0	0	0	0	0	10	0	0	0	0	10	0
	**8**	0	10	0	0	0	0	0	1	8	1	0	0	0	0	4	6	0	0
	**9**	0	8	2	0	0	0	0	0	3	7	0	0	9	1	0	0	0	0
	**10**	0	0	0	0	7	3	0	0	0	0	4	6	0	0	0	2	8	0
	**11**	0	10	0	0	0	0	0	0	7	3	0	0	0	0	9	1	0	0
	**12**	0	1	9	0	0	0	0	0	1	9	0	0	0	0	0	6	4	0
	**13**	0	0	8	2	0	0	0	0	0	0	4	6	0	0	0	0	10	0
	**Median**	**10**						**8**						**9**					
	**95% CI**	**Lower**	9		**Upper**	13		**Lower**	6		**Upper**	9		**Lower**	7		**Upper**	10	

Seat Positions		Front-Right-Seat						Rear-Right-Seat						Rear-Middle-Seat					

DRS (Gray Step)		11.0	10.0	9.0	8.0	7.0	6.0	11.0	10.0	9.0	8.0	7.0	6.0	11.0	10.0	9.0	8.0	7.0	6.0
**Dark (L2)/PG Camera**	**Total Votes**	**0**	**1**	**23**	**75**	**28**	**3**	**0**	**0**	**19**	**61**	**25**	**25**	**0**	**20**	**16**	**34**	**60**	**0**
	**Evaluator 1**	0	0	0	2	8	0	0	0	10	0	0	0	0	0	10	0	0	0
	**2**	0	0	10	0	0	0	0	0	0	1	9	0	0	0	0	1	9	0
	**3**	0	0	2	7	1	0	0	0	0	10	0	0	0	0	0	2	8	0
	**4**	0	0	0	10	0	0	0	0	0	10	0	0	0	10	0	0	0	0
	**5**	0	0	4	6	0	0	0	0	0	10	0	0	0	0	0	2	8	0
	**6**	0	0	1	8	1	0	0	0	0	10	0	0	0	0	0	2	8	0
	**7**	0	0	0	1	6	3	0	0	0	0	0	10	0	0	0	3	7	0
	**8**	0	0	0	9	1	0	0	0	7	3	0	0	0	0	0	10	0	0
	**9**	0	0	1	7	2	0	0	0	0	5	5	0	0	10	0	0	0	0
	**10**	0	0	0	2	8	0	0	0	0	0	0	10	0	0	0	2	8	0
	**11**	0	0	1	9	0	0	0	0	0	4	6	0	0	0	6	4	0	0
	**12**	0	0	3	7	0	0	0	0	2	7	1	0	0	0	0	6	4	0
	**13**	0	1	1	7	1	0	0	0	0	1	4	5	0	0	0	2	8	0
	**Median**	**8**						**8**						**7**					
	**95% CI**	**Lower**	7		**Upper**	8		**Lower**	6.5		**Upper**	8		**Lower**	7		**Upper**	9	

Seat Positions		Front-Right-Seat						Rear-Right-Seat						Rear-Middle-Seat					

DRS (Gray Step)		12.0	10.0	9.0	8.0	7.0	6.0	11.0	10.0	9.0	8.0	7.0	6.0	10.5	10.0	9.0	8.0	7.0	6.0
**Dark-BL (L3)/PG Camera**	**Total Votes**	**11**	**27**	**58**	**12**	**20**	**2**	**0**	**20**	**39**	**29**	**27**	**15**	**0**	**9**	**11**	**39**	**52**	**19**
	**Evaluator 1**	9	1	0	0	0	0	0	10	0	0	0	0	0	0	0	10	0	0
	**2**	0	0	0	4	6	0	0	0	0	0	10	0	0	0	0	3	7	0
	**3**	0	0	10	0	0	0	0	0	2	8	0	0	0	0	0	2	8	0
	**4**	0	10	0	0	0	0	0	10	0	0	0	0	0	0	10	0	0	0
	**5**	0	0	10	0	0	0	0	0	2	8	0	0	0	0	0	4	6	0
	**6**	0	0	10	0	0	0	0	0	2	8	0	0	0	0	0	4	6	0
	**7**	0	0	6	2	2	0	0	0	0	0	3	7	0	0	0	0	3	7
	**8**	0	8	2	0	0	0	0	0	10	0	0	0	0	0	0	6	4	0
	**9**	1	6	3	0	0	0	0	0	4	4	2	0	0	9	1	0	0	0
	**10**	0	0	0	5	3	2	0	0	0	0	5	5	0	0	0	0	4	6
	**11**	0	2	8	0	0	0	0	0	10	0	0	0	0	0	0	10	0	0
	**12**	1	0	9	0	0	0	0	0	9	1	0	0	0	0	0	0	10	0
	**13**	0	0	0	1	9	0	0	0	0	0	7	3	0	0	0	0	4	6
	**Median**	**9**						**8**						**7**					
	**95% CI**	**Lower**	7.5		**Upper**	10		**Lower**	7		**Upper**	9		**Lower**	6		**Upper**	8	

Seat Positions		Front-Right-Seat						Rear-Right-Seat						Rear-Middle-Seat					

DRS (Gray Step)		15.0	10.0	9.0	8.0	7.0	6.0	18.0	10.0	9.0	8.0	7.0	6.0	20.0	10.0	9.0	8.0	7.0	6.0
**Sunset-RW (L4)/NK Camera**	**Total Votes**	**14**	**64**	**24**	**6**	**19**	**3**	**42**	**53**	**20**	**12**	**2**	**1**	**35**	**49**	**35**	**11**	**0**	**0**
	**Evaluator 1**	9	1	0	0	0	0	10	0	0	0	0	0	10	0	0	0	0	0
	**2**	0	0	0	0	10	0	0	0	4	5	1	0	0	1	7	2	0	0
	**3**	0	10	0	0	0	0	0	10	0	0	0	0	0	10	0	0	0	0
	**4**	0	10	0	0	0	0	10	0	0	0	0	0	10	0	0	0	0	0
	**5**	0	10	0	0	0	0	0	10	0	0	0	0	0	8	2	0	0	0
	**6**	0	10	0	0	0	0	0	10	0	0	0	0	0	8	2	0	0	0
	**7**	0	3	5	2	0	0	0	2	5	2	1	0	0	0	5	5	0	0
	**8**	4	6	0	0	0	0	10	0	0	0	0	0	0	8	2	0	0	0
	**9**	0	6	4	0	0	0	6	4	0	0	0	0	10	0	0	0	0	0
	**10**	0	0	4	4	2	0	0	6	4	0	0	0	0	3	7	0	0	0
	**11**	0	5	5	0	0	0	6	4	0	0	0	0	2	8	0	0	0	0
	**12**	1	3	6	0	0	0	0	5	4	1	0	0	3	3	4	0	0	0
	**13**	0	0	0	0	7	3	0	2	3	4	0	1	0	0	6	4	0	0
	**Median**	**10**						**10**						**10**					
	**95% CI**	**Lower**	8		**Upper**	10		**Lower**	9		**Upper**	18		**Lower**	9		**Upper**	20	

Seat Positions		Front-Right-Seat						Rear-Right-Seat						Rear-Middle-Seat					

DRS (Gray Step)		12.0	10.0	9.0	8.0	7.0	6.0	12.0	10.0	9.0	8.0	7.0	6.0	11.5	10.0	9.0	8.0	7.0	6.0
**Sunset-RW (L4)/PG Camera**	**Total Votes**	**0**	**12**	**46**	**17**	**49**	**6**	**2**	**44**	**25**	**57**	**1**	**1**	**20**	**36**	**20**	**53**	**1**	**0**
	**Evaluator 1**	0	10	0	0	0	0	2	8	0	0	0	0	0	10	0	0	0	0
	**2**	0	0	0	0	10	0	0	0	0	10	0	0	0	0	0	10	0	0
	**3**	0	0	10	0	0	0	0	0	0	10	0	0	0	10	0	0	0	0
	**4**	0	0	10	0	0	0	0	10	0	0	0	0	10	0	0	0	0	0
	**5**	0	0	10	0	0	0	0	0	0	10	0	0	0	8	2	0	0	0
	**6**	0	1	9	0	0	0	0	0	0	10	0	0	0	8	2	0	0	0
	**7**	0	0	0	2	8	0	0	3	3	4	0	0	0	0	0	10	0	0
	**8**	0	0	0	0	10	0	0	10	0	0	0	0	0	0	3	7	0	0
	**9**	0	0	5	5	0	0	0	5	5	0	0	0	10	0	0	0	0	0
	**10**	0	0	0	0	8	2	0	0	0	8	1	1	0	0	2	8	0	0
	**11**	0	0	0	2	8	0	0	5	5	0	0	0	0	0	10	0	0	0
	**12**	0	1	2	7	0	0	0	0	9	1	0	0	0	0	1	9	0	0
	**13**	0	0	0	1	5	4	0	3	3	4	0	0	0	0	0	9	1	0
	**Median**	**8**						**9**						**9**					
	**95% CI**	**Lower**	7		**Upper**	9		**Lower**	8		**Upper**	10		**Lower**	8		**Upper**	10	

**Mean of 18 Thresholds (Gray Step)**					**8.8**			**Worst-Case Scenario Threshold (Gray Step)**									**10**		

**Table 5:** Taxicab facial photo evaluation study: Lens Distortion (LD) Votes. Unit: Percentage (%).

Seat Positions		Front-Right-Seat						Rear-Right-Seat						Rear-Middle-Seat					

LD (%)		0.0	15.0	30.0	45.0	60.0	70.0	0.0	15.0	30.0	45.0	60.0	70.0	0.0	15.0	30.0	45.0	60.0	70.0
**Daylight (L1)/NK Camera**	**Total Votes**	**0**	**20**	**31**	**47**	**12**	**20**	**0**	**12**	**28**	**23**	**10**	**57**	**0**	**28**	**32**	**19**	**21**	**30**
	**Evaluator 1**	0	10	0	0	0	0	0	10	0	0	0	0	0	10	0	0	0	0
	**2**	0	0	2	8	0	0	0	0	10	0	0	0	0	0	10	0	0	0
	**3**	0	0	3	6	1	0	0	0	0	0	0	10	0	0	0	4	6	0
	**4**	0	0	0	10	0	0	0	0	0	10	0	0	0	0	10	0	0	0
	**5**	0	0	4	5	1	0	0	0	0	0	0	10	0	0	0	4	6	0
	**6**	0	0	5	4	1	0	0	0	0	0	0	10	0	0	0	4	6	0
	**7**	0	0	0	0	0	10	0	0	0	0	0	10	0	0	0	0	0	10
	**8**	0	0	0	0	0	10	0	0	0	0	0	10	0	0	0	0	0	10
	**9**	0	6	4	0	0	0	0	2	6	2	0	0	0	7	3	0	0	0
	**10**	0	0	1	3	6	0	0	0	1	2	7	0	0	0	0	7	3	0
	**11**	0	0	10	0	0	0	0	0	10	0	0	0	0	5	5	0	0	0
	**12**	0	4	1	4	1	0	0	0	1	9	0	0	0	6	4	0	0	0
	**13**	0	0	1	7	2	0	0	0	0	0	3	7	0	0	0	0	0	10
	**Median**	**45**						**60**						**45**					
	**95% CI**	**Lower**	30		**Upper**	60		**Lower**	30		**Upper**	75		**Lower**	15		**Upper**	75	

Seat Positions		Front-Right-Seat						Rear-Right-Seat						Rear-Middle-Seat					

LD (%)		0.0	15.0	30.0	45.0	60.0	70.0	0.0	15.0	30.0	45.0	60.0	70.0	0.0	15.0	30.0	45.0	60.0	70.0
**Daylight (L1)/PG Camera**	**Total Votes**	**0**	**18**	**37**	**45**	**5**	**25**	**0**	**10**	**27**	**31**	**37**	**25**	**0**	**25**	**36**	**32**	**7**	**30**
	**Evaluator 1**	0	10	0	0	0	0	0	10	0	0	0	0	0	10	0	0	0	0
	**2**	0	0	0	10	0	0	0	0	10	0	0	0	0	0	10	0	0	0
	**3**	0	0	4	5	1	0	0	0	0	0	10	0	0	0	0	8	2	0
	**4**	0	0	0	10	0	0	0	0	0	10	0	0	0	0	10	0	0	0
	**5**	0	0	4	5	1	0	0	0	0	0	10	0	0	0	0	8	2	0
	**6**	0	0	5	4	1	0	0	0	0	0	10	0	0	0	0	8	2	0
	**7**	0	0	0	0	0	10	0	0	0	0	0	10	0	0	0	0	0	10
	**8**	0	0	0	0	0	10	0	0	0	0	0	10	0	0	0	0	0	10
	**9**	0	8	2	0	0	0	0	0	6	4	0	0	0	5	5	0	0	0
	**10**	0	0	0	3	2	5	0	0	0	7	3	0	0	0	1	8	1	0
	**11**	0	0	10	0	0	0	0	0	10	0	0	0	0	10	0	0	0	0
	**12**	0	0	6	4	0	0	0	0	1	9	0	0	0	0	10	0	0	0
	**13**	0	0	6	4	0	0	0	0	0	1	4	5	0	0	0	0	0	10
	**Median**	**45**						**45**						**45**					
	**95% CI**	**Lower**	30		**Upper**	68		**Lower**	30		**Upper**	68		**Lower**	23		**Upper**	75	

Seat Positions		Front-Right-Seat						Rear-Right-Seat						Rear-Middle-Seat					

LD (%)		0.0	15.0	30.0	45.0	60.0	70.0	0.0	15.0	30.0	45.0	60.0	70.0	0.0	15.0	30.0	45.0	60.0	70.0
**Dark (L2)/PG Camera**	**Total Votes**	**5**	**8**	**38**	**33**	**17**	**29**	**0**	**10**	**26**	**24**	**44**	**26**	**0**	**29**	**26**	**13**	**9**	**53**
	**Evaluator 1**	0	0	10	0	0	0	0	10	0	0	0	0	0	10	0	0	0	0
	**2**	0	2	6	2	0	0	0	0	10	0	0	0	0	0	10	0	0	0
	**3**	0	0	0	5	4	1	0	0	0	0	10	0	0	0	0	0	2	8
	**4**	0	0	0	0	0	10	0	0	0	10	0	0	0	0	10	0	0	0
	**5**	0	0	0	7	3	0	0	0	0	0	10	0	0	0	0	0	2	8
	**6**	0	0	0	4	5	1	0	0	0	0	10	0	0	0	0	0	2	8
	**7**	0	0	0	0	0	10	0	0	0	0	0	10	0	0	0	0	0	10
	**8**	0	0	0	3	3	4	0	0	0	0	0	10	0	0	0	0	0	10
	**9**	0	2	2	4	2	0	0	0	2	6	2	0	0	9	1	0	0	0
	**10**	0	1	3	3	0	3	0	0	4	3	3	0	0	0	0	8	2	0
	**11**	0	0	10	0	0	0	0	0	10	0	0	0	0	10	0	0	0	0
	**12**	5	1	2	2	0	0	0	0	0	5	5	0	0	0	5	5	0	0
	**13**	0	2	5	3	0	0	0	0	0	0	4	6	0	0	0	0	1	9
	**Median**	**45**						**52.5**						**45**					
	**95% CI**	**Lower**	30		**Upper**	60		**Lower**	30		**Upper**	75		**Lower**	15		**Upper**	75	

Seat Positions		Front-Right-Seat						Rear-Right-Seat						Rear-Middle-Seat					

LD (%)		0.0	15.0	30.0	45.0	60.0	70.0	0.0	15.0	30.0	45.0	60.0	70.0	0.0	15.0	30.0	45.0	60.0	70.0
**Total Votes**		**0**	**19**	**22**	**48**	**18**	**23**	**0**	**11**	**36**	**21**	**37**	**25**	**0**	**30**	**32**	**8**	**12**	**48**
**Dark-BL (L3)/PG Camera**	**Evaluator 1**	0	10	0	0	0	0	0	10	0	0	0	0	0	10	0	0	0	0
	**2**	0	0	0	10	0	0	0	0	10	0	0	0	0	0	10	0	0	0
	**3**	0	0	0	6	4	0	0	0	0	0	10	0	0	0	0	0	4	6
	**4**	0	0	0	10	0	0	0	0	10	0	0	0	0	0	10	0	0	0
	**5**	0	0	0	6	4	0	0	0	0	0	10	0	0	0	0	0	4	6
	**6**	0	0	0	5	5	0	0	0	0	0	10	0	0	0	0	0	4	6
	**7**	0	0	0	0	0	10	0	0	0	0	0	10	0	0	0	0	0	10
	**8**	0	0	0	0	0	10	0	0	0	0	0	10	0	0	0	0	0	10
	**9**	0	6	4	0	0	0	0	1	2	6	1	0	0	8	2	0	0	0
	**10**	0	0	0	2	5	3	0	0	4	6	0	0	0	0	2	8	0	0
	**11**	0	0	10	0	0	0	0	0	10	0	0	0	0	10	0	0	0	0
	**12**	0	3	6	1	0	0	0	0	0	9	1	0	0	2	8	0	0	0
	**13**	0	0	2	8	0	0	0	0	0	0	5	5	0	0	0	0	0	10
	**Median**	**45**						**45**						**45**					
	**95% CI**	**Lower**	30		**Upper**	60		**Lower**	30		**Upper**	68		**Lower**	15		**Upper**	75	

Seat Positions		Front-Right-Seat						Rear-Right-Seat						Rear-Middle-Seat					

LD (%)		0.0	15.0	30.0	45.0	60.0	70.0	0.0	15.0	30.0	45.0	60.0	70.0	0.0	15.0	30.0	45.0	60.0	70.0
**Sunset-RW (L4)/NK Camera**	**Total Votes**	**0**	**17**	**34**	**39**	**19**	**21**	**11**	**1**	**35**	**12**	**41**	**30**	**0**	**30**	**31**	**9**	**20**	**40**
	**Evaluator 1**	0	10	0	0	0	0	10	0	0	0	0	0	0	10	0	0	0	0
	**2**	0	0	0	10	0	0	0	1	9	0	0	0	0	0	10	0	0	0
	**3**	0	0	2	4	4	0	0	0	0	0	10	0	0	0	0	0	6	4
	**4**	0	0	0	10	0	0	0	0	10	0	0	0	0	0	10	0	0	0
	**5**	0	0	1	5	4	0	0	0	0	0	10	0	0	0	0	0	6	4
	**6**	0	0	2	4	4	0	0	0	0	0	10	0	0	0	0	0	6	4
	**7**	0	0	0	0	0	10	0	0	0	0	0	10	0	0	0	0	0	10
	**8**	0	0	0	0	0	10	0	0	0	0	0	10	0	0	0	0	0	10
	**9**	0	3	6	1	0	0	0	0	4	6	0	0	0	9	1	0	0	0
	**10**	0	0	0	2	7	1	0	0	2	5	3	0	0	0	2	8	0	0
	**11**	0	1	9	0	0	0	0	0	10	0	0	0	0	10	0	0	0	0
	**12**	0	2	6	2	0	0	1	0	0	1	5	3	0	1	8	1	0	0
	**13**	0	1	8	1	0	0	0	0	0	0	3	7	0	0	0	0	2	8
	**Median**	**45**						**60**						**45**					
	**95% CI**	**Lower**	30		**Upper**	60		**Lower**	30		**Upper**	75		**Lower**	15		**Upper**	75	

Seat Positions		Front-Right-Seat						Rear-Right-Seat						Rear-Middle-Seat					

LD (%)		0.0	15.0	30.0	45.0	60.0	70.0	0.0	15.0	30.0	45.0	60.0	70.0	0.0	15.0	30.0	45.0	60.0	70.0
**Sunset-RW (L4)/PG Camera**	**Total Votes**	**0**	**15**	**29**	**39**	**22**	**25**	**1**	**10**	**26**	**25**	**38**	**30**	**0**	**32**	**34**	**4**	**28**	**32**
	**Evaluator 1**	0	10	0	0	0	0	0	10	0	0	0	0	0	10	0	0	0	0
	**2**	0	0	0	10	0	0	0	0	10	0	0	0	0	0	10	0	0	0
	**3**	0	0	0	2	6	2	0	0	0	0	9	1	0	0	0	0	10	0
	**4**	0	0	0	10	0	0	0	0	0	10	0	0	0	0	10	0	0	0
	**5**	0	0	0	3	6	1	0	0	0	0	9	1	0	0	0	0	8	2
	**6**	0	0	0	2	7	1	0	0	0	0	9	1	0	0	0	0	8	2
	**7**	0	0	0	0	0	10	0	0	0	0	0	10	0	0	0	0	0	10
	**8**	0	0	0	0	0	10	0	0	0	0	0	10	0	0	0	0	0	10
	**9**	0	2	8	0	0	0	0	0	4	6	0	0	0	10	0	0	0	0
	**10**	0	0	1	5	3	1	0	0	1	9	0	0	0	0	7	3	0	0
	**11**	0	0	10	0	0	0	0	0	10	0	0	0	0	10	0	0	0	0
	**12**	0	1	7	2	0	0	1	0	1	0	8	0	0	2	7	1	0	0
	**13**	0	2	3	5	0	0	0	0	0	0	3	7	0	0	0	0	2	8
	**Median**	**45**						**60**						**30**					
	**95% CI**	**Lower**	30		**Upper**	60		**Lower**	30		**Upper**	75		**Lower**	15		**Upper**	75	

**Mean of 18 Thresholds (%)**							**47.1**			**Worst-Case Scenario Threshold (%)**							**30**		

**Table 6:** Taxicab facial photo evaluation study: Motion Blur (MB) Votes. Unit: Milliseconds (mS).

Seat Positions		Rear Middle-Seat						Seat Positions		Rear-Middle-Seat					

MB (mS)		20	25	33	40	50	67	MB (mS)		20	25	33	40	50	67
**Daylight (L1)/NK Camera**	**Total Votes**	**5**	**48**	**36**	**30**	**4**	**7**	**Dark-BL (L3)/PG Camera**	**Total Votes**	**3**	**41**	**47**	**31**	**7**	**1**
	**Evaluator 1**	0	0	10	0	0	0		**Evaluator 1**	0	0	1	8	1	0
	**2**	0	0	2	7	1	0		**2**	0	7	3	0	0	0
	**3**	0	8	1	1	0	0		**3**	0	5	3	2	0	0
	**4**	0	0	10	0	0	0		**4**	0	0	7	3	0	0
	**5**	0	9	0	1	0	0		**5**	0	0	7	3	0	0
	**6**	0	8	2	0	0	0		**6**	0	5	3	2	0	0
	**7**	0	0	4	6	0	0		**7**	0	0	2	6	2	0
	**8**	0	0	0	0	3	7		**8**	0	2	5	2	1	0
	**9**	5	5	0	0	0	0		**9**	0	1	6	3	0	0
	**10**	0	1	4	5	0	0		**10**	0	6	1	2	1	0
	**11**	0	10	0	0	0	0		**11**	3	7	0	0	0	0
	**12**	0	7	3	0	0	0		**12**	0	7	3	0	0	0
	**13**	0	0	0	10	0	0		**13**	0	1	6	0	2	1
	**Median**	**95% CI-Lower**			**95% CI-Upper**				**Median**	**95% CI-Lower**			**95% CI-Upper**		
	**33.3**	25			40				**33.3**	25			33.3		

Seat Positions		Rear Middle-Seat						Seat Positions		Rear-Middle-Seat					

MB (mS)		20	25	33	40	50	67	MB (mS)		20	25	33	40	50	67
**Daylight (L1)/PG Camera**	**Total Votes**	**0**	**47**	**33**	**41**	**9**	**0**	**Sunset-RW (L4)/NK Camera**	**Total Votes**	**2**	**28**	**64**	**35**	**1**	**0**
	**Evaluator 1**	0	10	0	0	0	0		**Evaluator 1**	0	1	9	0	0	0
	**2**	0	0	5	5	0	0		**2**	0	0	3	7	0	0
	**3**	0	1	2	6	1	0		**3**	0	0	9	1	0	0
	**4**	0	10	0	0	0	0		**4**	0	0	10	0	0	0
	**5**	0	1	2	6	1	0		**5**	0	0	8	2	0	0
	**6**	0	1	2	6	1	0		**6**	0	0	8	2	0	0
	**7**	0	0	4	4	2	0		**7**	0	0	1	9	0	0
	**8**	0	0	2	6	2	0		**8**	0	3	6	1	0	0
	**9**	0	5	4	1	0	0		**9**	2	8	0	0	0	0
	**10**	0	3	6	1	0	0		**10**	0	0	5	5	0	0
	**11**	0	10	0	0	0	0		**11**	0	10	0	0	0	0
	**12**	0	6	3	1	0	0		**12**	0	6	4	0	0	0
	**13**	0	0	3	5	2	0		**13**	0	0	1	8	1	0
	**Median**	**95% CI-Lower**			**95% CI-Upper**				**Median**	**95% CI-Lower**			**95% CI-Upper**		
	**36.7**	25			40				**33.3**	25			40		

Seat Positions		Rear Middle-Seat						Seat Positions		Rear-Middle-Seat					

MB (mS)		20	25	33	40	50	67	MB (mS)		20	25	33	40	50	67
**Dark (L2)/PG Camera**	**Total Votes**	**7**	**43**	**38**	**42**	**0**	**0**	**Sunset-RW (L4)/PG Camera**	**Total Votes**	**4**	**30**	**50**	**33**	**13**	**0**
	**Evaluator 1**	0	10	0	0	0	0		**Evaluator 1**	0	4	6	0	0	0
	**2**	0	0	4	6	0	0		**2**	0	2	8	0	0	0
	**3**	0	2	3	5	0	0		**3**	0	0	4	5	1	0
	**4**	0	0	10	0	0	0		**4**	0	0	10	0	0	0
	**5**	0	2	3	5	0	0		**5**	0	0	4	5	1	0
	**6**	0	2	3	5	0	0		**6**	0	0	4	5	1	0
	**7**	0	0	1	9	0	0		**7**	0	0	0	5	5	0
	**8**	0	3	6	1	0	0		**8**	0	0	7	3	0	0
	**9**	3	7	0	0	0	0		**9**	3	7	0	0	0	0
	**10**	0	1	8	1	0	0		**10**	0	1	4	5	0	0
	**11**	3	7	0	0	0	0		**11**	1	9	0	0	0	0
	**12**	1	9	0	0	0	0		**12**	0	7	3	0	0	0
	**13**	0	0	0	10	0	0		**13**	0	0	0	5	5	0
	**Median**	**95% CI-Lower**			**95% CI-Upper**				**Median**	**95% CI-Lower**			**95% CI-Upper**		
	**33.3**	25			40				**33.3**	25			40		

**Mean of 6 Thresholds (mS)**					**33.9**			**Worst-Case Scenario Threshold (mS)**					**33.3**		

**Table 7:** Summary of median and worst-case scenario thresholds and 95% confidence intervals (Head-Height = 30 cm).

Seat Positions			Front-Right-Seat			Rear-Right-Seat			Rear-Middle-Seat		

Thresholds		Units	Median	95% CI Lower	95% CI Higher	Median	95% CI Lower	95% CI Higher	Median	95% CI Lower	95% CI Higher
Daylight (L1)/NK Camera	Photograph Resolution	LPHH	56.2	56.2	75.2	56.1	56.1	83.2	72.7	54.3	168.7
	Dynamic Range (Highlight)	Gray Step	4.5	4	6	6	4	6	5	4	5
	Dynamic Range (Shadow)	Gray Step	9.5	9	10	8	8	10	10	8	10
	Lens Distortion	%	45	30	60	60	30	75	45	15	75
	Motion Blur	mS							33.3	25	40

Seat Positions			Front-Right-Seat			Rear-Right-Seat			Rear-Middle-Seat		

Thresholds		Units	Median	95% CI Lower	95% CI Higher	Median	95% CI Lower	95% CI Higher	Median	95% CI Lower	95% CI Higher
Daylight (L1)/PG Camera	Photograph Resolution	LPHH	61.7	59.9	61.7	55.4	55.4	110.1	69.6	58.5	93.7
	Dynamic Range (Highlight)	Gray Step	4	2.5	5	5	4	5.5	4	4	5
	Dynamic Range (Shadow)	Gray Step	10	9	13	8	6	9	9	7	10
	Lens Distortion	%	45	30	67.5	45	30	67.5	45	22.5	75
	Motion Blur	mS							36.7	25	40

Seat Positions			Front-Right-Seat			Rear-Right-Seat			Rear-Middle-Seat		

Thresholds		Units	Median	95% CI Lower	95% CI Higher	Median	95% CI Lower	95% CI Higher	Median	95% CI Lower	95% CI Higher
Dark (L2)/PG Camera	Photograph Resolution	LPHH	60.9	52.4	60.9	55	55	93.6	120.5	51.7	120.5
	Dynamic Range (Highlight)	Gray Step	4.5	4	5	3	2.5	4	4	3	4
	Dynamic Range (Shadow)	Gray Step	8	7	8	8	6.5	8	7	7	9
	Lens Distortion	%	45	30	60	52.5	30	75	45	15	75
	Motion Blur	mS							33.3	25	40

Seat Positions			Front-Right-Seat			Rear-Right-Seat			Rear-Middle-Seat		

	Thresholds	Units	Median	95% CI Lower	95% CI Higher	Median	95% CI Lower	95% CI Higher	Median	95% CI Lower	95% CI Higher
Dark-BL (L3)/PG Camera	Photograph Resolution	LPHH	72.9	55.9	89.9	49.6	49.6	71.5	47.4	47.4	96.9
	Dynamic Range (Highlight)	Gray Step	4	3	5	4	3	4	3	3	5
	Dynamic Range (Shadow)	Gray Step	9	7.5	10	8	7	9	7	6	8
	Lens Distortion	%	45	30	60	45	30	67.5	45	15	75
	Motion Blur	mS							33.3	25	33.3

Seat Positions			Front-Right-Seat			Rear-Right-Seat			Rear-Middle-Seat		

Thresholds		Units	Median	95% CI Lower	95% CI Higher	Median	95% CI Lower	95% CI Higher	Median	95% CI Lower	95% CI Higher
Sunset-RW (L4)/NK Camera	Photograph Resolution	LPHH	57.2	57.2	72.8	53.8	53.8	95.2	52.8	52.8	118.8
	Dynamic Range (Highlight)	Gray Step	4	3	5	5.5	4.5	6	5	5	6
	Dynamic Range (Shadow)	Gray Step	10	8	10	10	9	18	10	9	20
	Lens Distortion	%	45	30	60	60	30	75	45	15	75
	Motion Blur	mS							33.3	25	40

Seat Positions			Front-Right-Seat			Rear-Right-Seat			Rear-Middle-Seat		

Thresholds		Units	Median	95% CI Lower	95% CI Higher	Median	95% CI Lower	95% CI Higher	Median	95% CI Lower	95% CI Higher
Sunset-RW (L4)/PG Camera	Photograph Resolution	LPHH	46.5	46.5	93.6	87.1	55.7	108.5	55.6	55.6	126.1
	Dynamic Range (Highlight)	Gray Step	3	2	4	4	2	5	5	4	5
	Dynamic Range (Shadow)	Gray Step	8	7	9	9	8	10	9	8	10
	Lens Distortion	%	45	30	60	60	30	75	30	15	75
	Motion Blur	mS							33.3	25	40

Mean and Worst-Case Scenario Photograph Thresholds	Unit	Mean	Worst-Case Scenario
Eighteen Photograph Resolution Thresholds	LPHH	62.8	88.2*
Eighteen Dynamic Range (Highlight) Thresholds	Gray Step	4.3	3
Eighteen Dynamic Range (Shadow) Thresholds	Gray Step	8.8	10
Eighteen Lens Distortion Thresholds	%	47.1	30
Six Motion Blur Thresholds	mS	33.9	33.3

* The worst-case scenario condition resolution threshold = (120.5 × 69 + 51.7 ×61)/(69 + 61) = 88.2 LPHH (see discussion in Section 4.1)
